# ZC3H13 mediates N6-methyladenosine modification of SNTB1 to promote epithelial-mesenchymal transition in gastric cancer

**DOI:** 10.1038/s41419-025-07889-2

**Published:** 2025-08-07

**Authors:** Xiaozhou Xie, Yulong Zhao, Jin Liu, Han Li, Xingyu Zhu, Yuan Liu, Yaodong Sang, Kang Xu, Fengying Du, Huicheng Ren, Xiaoling Cui, Baoshan Cai, Hao Chen, Nanping Wu, Guihua Hou, Changqing Jing, Wei Chong, Leping Li, Liang Shang

**Affiliations:** 1 Department of Gastrointestinal Surgery, Shandong Provincial Hospital Affiliated to Shandong First Medical University, Jinan, China; 2 Shandong Provincial Laboratory of Translational Medicine Engineering for Digestive Tumors, Shandong Provincial Hospital, Jinan, China; 3 Medical Science and Technology Innovation Center, Shandong First Medical University & Shandong Academy of Medical Sciences, Jinan, China; 4 Cell Biology Research Platform, Jinan Microecological Biomedicine Shandong Laboratory, Jinan, China; 5 Research Center for Experimental Nuclear Medicine, School of Basic Medical Sciences, Shandong University, Jinan, China; 6 Department of Gastroenterology, Shandong Provincial Hospital Affiliated to Shandong First Medical University, Jinan, China; 7 Department of Gastrointestinal Surgery, The First Affiliated Hospital of Shandong First Medical University, Jinan, China; 8 Department of Gastrointestinal Surgery, Zibo Central Hospital, Zibo, China; 9 Clinical Research Center of Shandong University, Clinical Epidemiology Unit, Qilu Hospital of Shandong University, Jinan, China

**Keywords:** Cancer, Gastric cancer

## Abstract

Gastric cancer (GC) is one of the highly aggressive human malignant tumors. However, as one of the components of m6A methylation, ZC3H13 has not been reported to regulate the occurrence and development of GC. We determined the expression of ZC3H13 in GC and its correlation with prognosis using the TCGA and ACRG datasets. The effect of ZC3H13 on GC was elucidated through in vivo and in vitro assays. Next, we identified the downstream target of ZC3H13 via MeRIP-seq, and RNA-seq combined with multi-omics analysis, and explored the regulatory mechanism of ZC3H13 in GC using methods such as immunoprecipitation. We found that ZC3H13 is highly expressed in GC tissues and contributes to the poor prognosis of GC patients. ZC3H13 promoted the proliferation, invasion and migration of GC cells in vivo and in vitro, possibly by facilitating the EMT process. In addition, SNTB1 was identified as a downstream target of ZC3H13. ZC3H13-regulated YTHDF1-dependent m6A modification led to post-transcriptional activation of SNTB1 and induce phenotypic changes and EMT activation in GC cells. We determined that ZC3H13 is significantly upregulated in GC and promotes its progression. ZC3H13 mediated YTHDF1-dependent m6A modification of SNTB1 promotes the progression of GC by influencing the EMT process. Our study is the first to report the crucial role of ZC3H13 mediated m6A modification in GC, and we believe that ZC3H13 can serve as a potential therapeutic target for GC.

## Introduction

Gastric cancer (GC) is one of the highly aggressive human malignant tumors, and despite advances in endoscopic techniques that have led to a decrease in its incidence, GC remains the fourth leading cause of cancer-related death due to limited treatment methods [1, 2]. Despite continuous improvements in diagnostic and therapeutic methods, the prognosis of metastatic GC remains poor, and the mortality rate remains high [3]. Epithelial-mesenchymal transition (EMT) is one of the important molecular steps of cancer progression, and leads to cytoskeletal remodeling of tumor cells, altered morphology, and weakened intercellular adhesion, leading to tumor invasion and metastasis, which in turn leads to poor prognosis [4, 5]. Loss of E-cadherin attachment is thought to be an early event in EMT, and Vimentin is thought to be a key molecule in EMT-mediated tumor metastasis. During the EMT process, the expression of the mesenchymal markers N-cadherin and Vimentin is increased. β-catenin is isolated from the cadherin complex and translocated into the nucleus, via simultaneous hydrolysis of collagen components in the extracellular matrix (ECM) by matrix metalloproteinases (MMPs) [6, 7]. An in-depth understanding the mechanism of EMT in the progression of GC can improve treatment strategies and patient prognosis.

N6-methyladenosine (m6A) refers to the methylation of the sixth nitrogen atom of adenylate, which is the most common modification in eukaryotic mRNAs [8]. The m6A modifications are mediated by three classes of enzymes, writers, erasers, and readers, which regulate RNA processing and export via posttranscriptional modifications, modulate RNA stability and affect RNA translation [9–11]. The typical m6A methyltransferase complex is composed mainly of METTL3, METTL14, and WTAP. Zinc finger CCCH-type containing 13 (ZC3H13) regulates m6A methylation by inducing the nuclear localization of the ZC3H13-WTAP-Virilizer-Hakai complex [12, 13]. Published studies have shown that m6A methylation plays an important role in human cancers. For example, METTL3-mediated m6A modification promotes EMT and metastasis in GC [14]. KIAA1429 activates the JNK/MAPK pathway in a m6A-dependent manner to promote tumorigenesis and gefitinib resistance in lung adenocarcinoma [15]. The demethylase ALKBH5 inhibits GC invasion through m6A modification of PKMYT1. The methyltransferase ZC3H13 has also been reported to play a role in the progression of a variety of cancers, including cervical [16], colorectal [17], pancreatic [18], hepatocellular [19], thyroid [20] and breast cancer [21]. However, the role of ZC3H13 in GC has not been reported.

In the present study, we demonstrated that the methyltransferase ZC3H13 promotes GC proliferation and affects GC migration and invasion by promoting EMT, and that this effect is achieved by modifying the downstream target gene syntrophin beta 1 (SNTB1) with m6A. In conclusion, we believe that ZC3H13 plays an important role in GC progression and can be used as a promising biomarker to predict the prognosis of GC patients.

## Methods and material

### Clinical samples

Obtaining GC tissue samples through surgery from Shandong Provincial Hospital Affiliated to Shandong First Medical University. All patients were diagnosed with EC for the first time, and none had any other malignancies. The study protocol was approved by the Ethics Committee of Shandong Provincial Hospital Affiliated to Shandong First Medical University (NSFC: NO.2022-875). All included respondents signed the informed consent. All experiments involving human samples were conducted following the *Declaration of Helsinki*.

### Immunohistochemistry (IHC)

The tissue sections were placed in a 65 °C roaster for more than 2 h, deparaffinized in xylene, hydrated in gradient ethanol, and subsequently antigenically repaired using sodium citrate in an autoclave. The membranes were permeabilized with 0.3% Triton X-100 (Solarbio, T8200) followed by closure with 5% goat serum (Solarbio, SL038), and the sections were incubated with the primary antibody at 4 °C overnight. The expression of target proteins was detected after secondary antibody incubation with 3,3′-diaminobenzidine (DAB) solution according to the manufacturer’s instructions (ZSGB-BIO, Beijing, China). Independent scoring was performed by two professional pathologists in the following manner (immunoreaction score, IRS): The intensity of cell staining was graded into 4 levels, with no positive staining (negative) scoring 0, yellowish (weakly positive) scoring 1, brown-yellow (positive) scoring 2, and brown (strongly positive) scoring 3. The percentage of positive cells was graded into 4 levels, with ≤25% scoring 1, 26–50% scoring 2, 51–75% scoring 3, and >75% scoring 4, and the two scores were multiplied to arrive at the final scoring result. For the 60 tissue microarrays, we used ImageJ software with the IHC Profiler plug-in installed for the calculation of staining positivity [22]. The following four scores were given: high positive (3+), positive (2+), low positive (1+), and negative (0), which were multiplied by the corresponding percentage of the staining area and summed to obtain the final score.

### Cell culture and transfection

The human gastric mucosa cell line (GES-1), human renal epithelial cell line (293T) and human STAD cell lines (AGS, MKN-45, HGC-27, and MKN-28) were purchased from Cyanen Biosciences (Guangzhou, China), all with original lineage tracing and STR (Short Tandem Repeat) identification reports. The gene typing of the cell lines was correct, and the mycoplasma detection was negative, and cultured at 37 °C with 5% CO2. The GES-1 and 293 T cells were cultured in Dulbecco’s modified Eagle medium (DMEM) (Gibco, Grand Island, USA) supplemented with 10% fetal bovine serum (FBS) (Umedium, He Fei, China) and 1% penicillin‒streptomycin (10 U/mL, Thermo Fisher). The AGS, MKN-45, HGC-27, and MKN-28 cells were cultured in RPMI 1640 culture medium (Gibco, Grand Island, USA) under the same conditions as the GES-1 cells. Lentiviruses for the knockdown and overexpression of ZC3H13 and its negative control were purchased from Genechem (Shanghai, China), and the cells were selected with puromycin (Beyotime, ST551) and G418 (MCE, HY-K1056) after transfection according to the manufacturer’s instructions. Small interfering RNA (siRNA) and a negative control were purchased from GenePharma (Suzhou, China). Lipofectamine 2000 (Thermo Fisher) was used to produce stable transfectants, and the expression efficiency was tested 48 h after transfection.

### Quantitative real-time PCR (qRT-PCR)

Total RNA was extracted using RNA-easy Isolation Reagent (Vazyme, China) and cDNA was obtained using a reverse transcription kit (Yugong Biolabs, China). The qRT-PCR was conducted with Taq SYBR Green qPCR Premix (Yugong Biolabs, China) and an Applied Biosystems QuantStudio 1 Real-Time PCR System (Applied Biosystems, ThermoFish) to detect gene expression. Relative gene expression was calculated by the 2^-ΔΔCt^ method, with GAPDH used as an endogenous control.

### Western blotting

The cells were lysed on ice using RIPA lysis buffer and then centrifuged at 12,000 × *g* for 30 min at 4 °C. Proteins were separated using sodium dodecyl sulfate-polyacrylamide gel electrophoresis (SDS-PAGE) and transferred to polyvinylidene fluoride (PVDF) membranes. The primary antibodies targeting β-actin (Proteintech, 20536-1-AP), ZC3H13 (Sigma, HPA040140), SNTB1 (GeneTex, GTX132898), E-cadherin (Proteintech, 20874-1-AP), N-cadherin (Proteintech, 66219-1-Ig), Vimentin (Proteintech, 60330-1-Ig), β-catenin (Proteintech, 51067-2-AP), FGD4 (GeneTex, GTX109859), IGF2BP1 (Proteintech, 22803-1-AP), IGF2BP2 (Proteintech, 11601-1-AP), YTHDC2 (Proteintech, 27779-1-AP), and YTHDF1 (Diagbio, db14438) were incubated overnight at 4 °C after blocking with 5% skim milk, and the secondary antibodies were incubated at room temperature for 1 h before the protein bands were visualized via a western blot imaging system (AMERSHAM ImageQuant 800).

### CCK-8 assays

Cell proliferation ability was detected via the Cell Counting Kit-8 (CCK8) (Dojindo, Japan), and the cell proliferation rate was calculated by measuring the optical density (OD) value of each well at a fixed time every day for 5 days.

### Colony formation

The cells were inoculated into 6-well plates at a density of 1000 cells per well, and the medium was changed periodically. After 2 weeks, the cells were fixed with 4% paraformaldehyde and stained with crystal violet, and the number of clones was calculated via ImageJ after images were taken.

### Wound healing assays

The cells were inoculated into 6-well plates, and three wounds per well were made using the pipette tip at > 95% cell fusion. Photographs of the same microscope field of view of each wound at 0 h and 48 h were taken. The ratio of the cell migration area to the wound area at 0 h was the cell migration rate. Three independent experiments were conducted to obtain statistical data for quantification.

### Transwell assays

For the migration experiments, 200 μL of basal medium containing 6 × 10^4^ cells was inoculated into the top chamber (Corning). For the invasion experiments, 200 μL of basal medium containing 1 × 10^5^ cells was inoculated into the top chamber, which was coated with Matrigel. A total of 600 μL of complete medium containing 10% FBS was added to the bottom chamber. After incubation for 24 h, the cells in the top chamber were fixed with 4% paraformaldehyde and stained with crystal violet dye. After being washed with PBS, the cells that did not invade the lower side of the chamber were removed via a cotton swab and counted under a microscope.

### Methylated RNA immunoprecipitation (MeRIP)

Assays were performed using a MeRIP kit (BersinBio, Guangzhou, China). Total RNA was extracted from the STAD cells using TRIzol reagent, fragmented according to the manufacturer’s instructions, and immunoprecipitated using m6A and IgG antibodies. The RNA was enriched via magnetic beads and reverse transcribed to cDNA after elution and purification. The PCR amplification of possible modified fragments was performed using specific primers, detected by DNA gel electrophoresis and analyzed via qRT-PCR.

### Luciferase reporter assays

293T cells in 24-well plates were transfected with a luciferase reporter gene, and all cells were harvested 48 h posttransfection and analyzed using the Dual Luciferase Reporter Gene Assay System (Promega, USA, Cat. #E1960). The relative ratio of firefly luciferase activity to sea kidney luciferase activity was determined.

### RNA stability assays

AGS cells were inoculated in 6-well plates and treated with 5 μg/mL actinomycin D (MCE, HY-17559) for different durations the following day. Total RNA was then extracted using RNA-easy Isolation Reagent (Vazyme, China), analyzed via qRT-PCR and normalized to GAPDH.

### RNA pull-down assays

Two micrograms of biotin-containing probes generated by in vitro transcription of the SNTB1 CDS region and 3’UTR were taken, 100 μL of RNA structure buffer was added, and the mixture was incubated at room temperature for 30 min. Twenty microlitres of magnetic beads were incubated with the refolding probe at 4 °C by spin shaking for 2 h. The magnetic beads was washed lightly with Tris-Triton lysis buffer once, and then, cell lysis buffer was added and incubated by spin shaking at 4 °C for 4 h. The magnetic beads were washed 10 times with Tris-Triton (containing protease and RNAse inhibitor), loading buffer was added, and the mixture was immersed in a metal bath at 100 °C for 10 min. After the magnetic beads were removed, a western blotting assay was performed.

### RNA immunoprecipitation (RIP) assays

Forty microlitres of magnetic beads were removed, washed twice with PBS, and then incubated overnight with PBS-diluted YTHDF1 antibody by spin shaking at 4 °C. IgG was used as a control. The cell lysate or normal IgG was incubated with magnetic beads at 4 °C for 6 h by spin shaking, after which the beads were washed 8 times with Tris-Triton. The above magnetic beads antibody mixture was resuspended in 200 μL of proteinase K buffer (containing 20 µg of proteinase K) and incubated at 55 °C for 30 min. The RNA was extracted, the interaction between the YTHDF1 and SNTB1 transcripts was determined via qRT-PCR, and the results were normalized to the input.

### In vivo tumorigenesis assays

Four-week-old BALB/c male mice were purchased from Beijing Vital River Laboratory Animal Technology Co., Ltd. (Beijing, China). The subcutaneous xenograft tumor models were established using HGC-27 and MKN-45 cells. A total of 2 × 10^6^ HGC-27 cells and 3 × 10^6^ MKN-45 cells were injected into the anterior flank of each mouse (Random grouping, with n = 5 in each group). The longest and shortest diameters of the tumors were measured every 3 days after tumor formation, and the tumor volume (V) was calculated according to the following formula: V = (the longest diameter × the shortest diameter^2^)/2. Lung and liver metastasis models were established using MKN-45 cells. For the lung metastasis model, 2 × 10^6^ cells per mouse were injected via the tail vein into mice (Random grouping, with n = 5 in each group). After 4 weeks, the mice were euthanized and dissected to observe lung metastasis. For the liver metastasis model, after sterilizing the abdominal skin of the mice on a sterile bench, surgery was performed to expose the spleen of mice. A total of 4 × 10^6^ cells were injected into the spleen of each mouse (Random grouping, with n = 5 in each group). Following this, the wounds were sutured and the area was disinfected. Five weeks later, the mice were euthanized, and the livers were dissected to observe the metastasis. The dissected lungs and livers were subjected to paraffin embedding, followed by hematoxylin and eosin (H&E) staining.

All operations of this study were approved by the Animal Ethics Committee of Shandong First Medical University Affiliated Provincial Hospital (NSFC: NO.2022-875).

### Statistical analysis

Bioinformatics-related statistical analyses were performed using an online database or R language software (R-4.2.2,64-bit), survival analysis was performed using the “survival” package, and GO and KEGG data visualization was performed using the “ggplot2” package.

For the cellular and molecular biology experiments, SPSS and Prism 8 (GraphPad) were used to analyze data from at least three independent experimental replicates using the t test or Wilcoxon rank-sum test; p < 0.05 was considered to indicate statistical significance.

## Results

### Up-regulated ZC3H13 expression is correlated with poor outcomes in GC patients

**Fig. 1:**
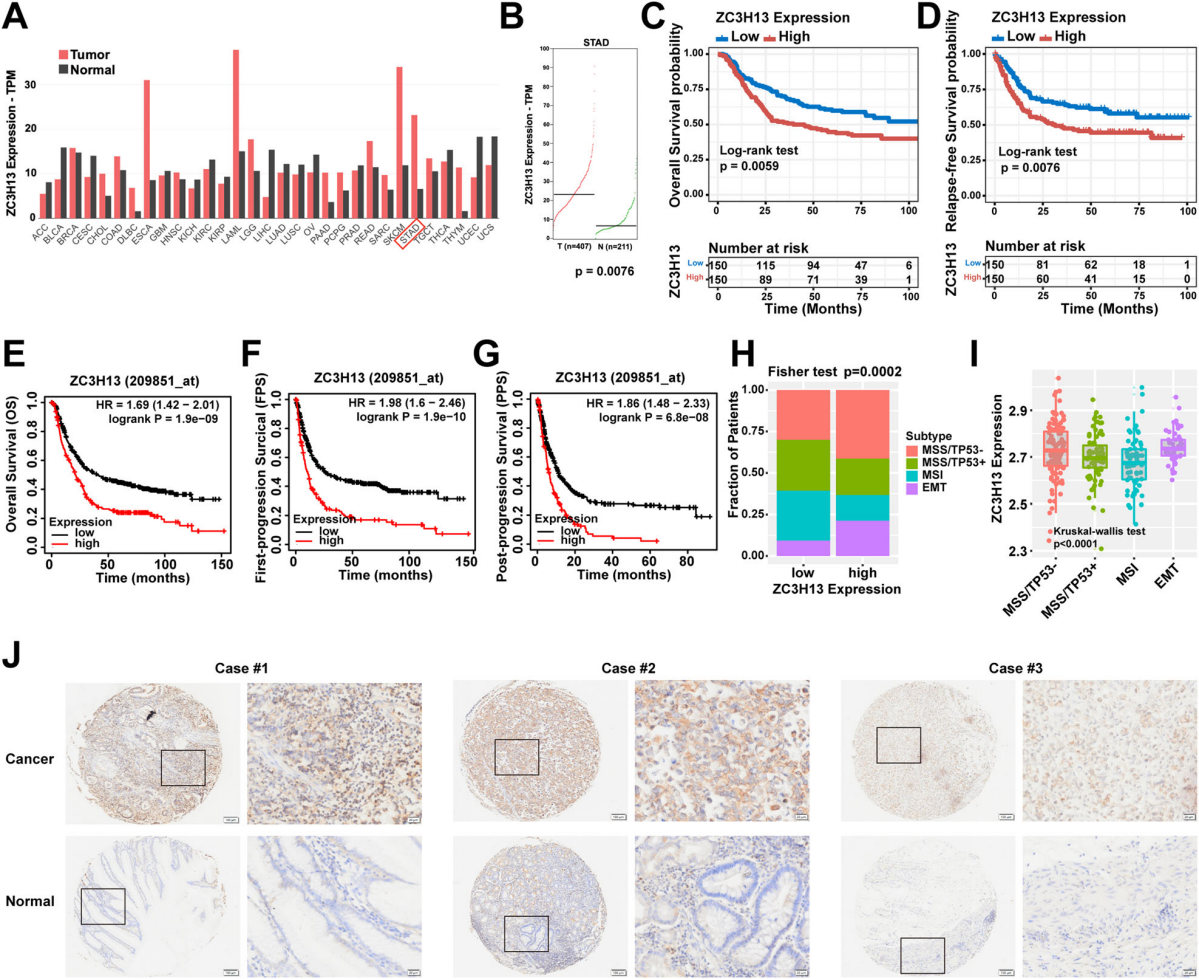
ZC3H13 was overexpressed in the GC tissues compared to the controls and is associated with poor prognosis. **A**, **B** ZC3H13 expression in the TCGA datasets. **C**–**G** Relationship between the expression of ZC3H13 and the prognosis of patients with GC. The data from the TCGA dataset revealed that ZC3H13 is highly expressed in the CIN (**H**) and EMT (**I**) groups. **J** Representative ZC3H13 expression in GC and adjacent tissues (magnification, 50× and 200×).

We obtained expression data for ZC3H13 in pan-cancer from the public database GEPIA and found that ZC3H13 expression is significantly elevated in STAD (Fig. 1A, B). We downloaded survival data for GC patients from the Asian Cancer Research Group (ACRG) and The Cancer Genome Atlas (TCGA) databases and found that patients with the overexpression of ZC3H13 had decreased overall survival (OS) (Fig. 1C) and relapse-free survival (RFS) (Fig. 1D). Based on the KM-plotter database, we further revealed that GC patients with high ZC3H13 expression had poorer OS, first-progression survival (FPS) and post-progression survival (PPS) (Fig. 1E–G). Analyzing GC sequencing data from the ACRG and TCGA databases in combination with molecular phenotyping revealed that ZC3H13 was more highly expressed in poorer prognostic subtypes [EMT and chromosomal instability (CIN) groups], which suggests that GC with high ZC3H13 expression have a greater degree of malignancy (Fig. 1H, I). We subsequently performed IHC staining using tissue microarrays containing 60 samples of GC and corresponding normal tissues, which revealed that the expression of ZC3H13 was significantly greater in GC tissues than in normal tissues (Fig. 1J).

### ZC3H13 knockdown inhibited the proliferation, migration, and invasion of GC cells in vitro

**Fig. 2:**
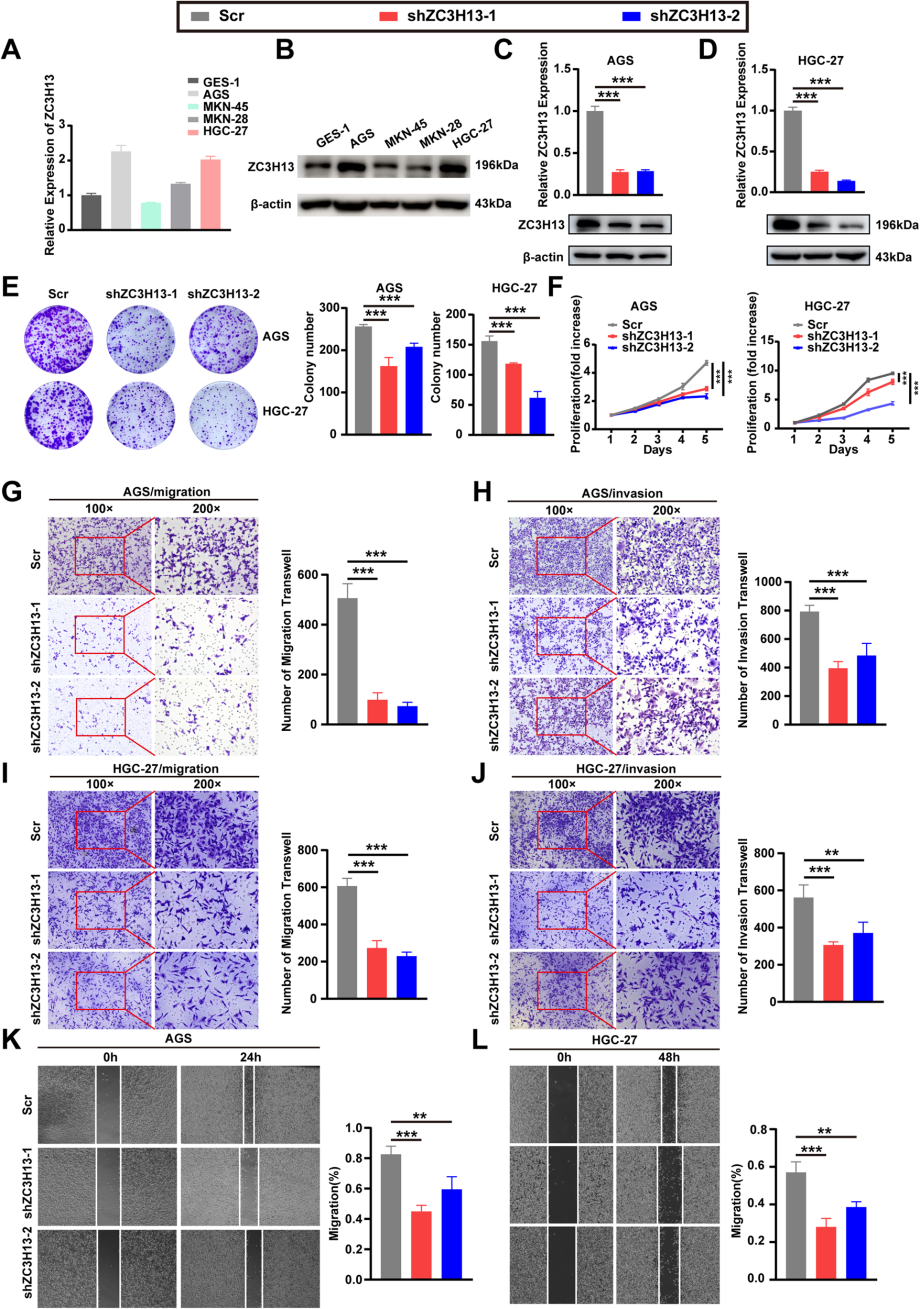
ZC3H13 knockdown inhibited the proliferation, invasion and migration of GC cells in vitro. **A**, **B** Endogenous expression of ZC3H13 in GC cell lines. ZC3H13 mRNA and protein expression decreased in AGS (**C**) and HGC-27 (**D**) cells after transfection with either shZC3H13-1 or shZC3H13-2. **E** ZC3H13 knockdown inhibited the proliferation capacity and colony formation of AGS and HGC-27 cells. **F** Down-expression of ZC3H13 inhibited the proliferative viability of AGS and HGC-27 cells. ZC3H13 knockdown inhibited the migration (**G**) and invasion (**H**) ability of AGS cells, as well as HGC-27 (**I**, **J**) cells. Wound healing assays revealed that ZC3H13 knockdown weakened the migration ability of AGS (**K**) and HGC-27 (**L**) cells. *P < 0.05, **P < 0.01, and ***P < 0.001 represent varying degrees of significance between the indicated groups.

To explore the function of ZC3H13 in GC, we assessed the expression level of ZC3H13 in GC cell lines. Compared with that in the human normal gastric mucosal epithelial cell line GES-1, ZC3H13 expression was significantly higher in the AGS and HGC-27 cell lines and lower in the MKN-45 and MKN-28 cell lines (Fig. 2A, B). We selected the AGS and HGC-27 cell lines to establish ZC3H13-silenced models and the MKN-45 cell line to establish a ZC3H13-overexpression model. Transfection of AGS and HGC-27 cells with lentivirus reduced the mRNA and protein expression of ZC3H13 in the cells (Fig. 2C, D). Compared with the control, ZC3H13 knockdown significantly inhibited the growth of AGS and HGC-27 cells in colony formation assays (Fig. 2E). Similarly, an inhibitory effect on the proliferative capacity of AGS and HGC-27 cells after the knockdown of ZC3H13 was demonstrated via CCK-8 assays (Fig. 2F). Transwell assays demonstrated that the knockdown of ZC3H13 significantly inhibited the migration and invasion ability of AGS and HGC-27 cells (Fig. 2G–J), and wound healing assays further revealed that ZC3H13 knockdown inhibited the migration ability of AGS and HGC-27 cells (Fig. 2K, L).

In addition, to exclude the off-target effect of shRNA and to further validate the effect of ZC3H13 knockdown on the migration and invasion ability of GC cells, we designed two new siRNA sequences and obtained better knockdown efficiency (Fig. S1A, B). And as shown in Fig. S1, knocking down ZC3H13 with siRNA significantly inhibited the migration and invasion ability of AGS cells.

### ZC3H13 overexpression promoted the proliferation, migration, and invasion of GC cells in vitro

**Fig. 3:**
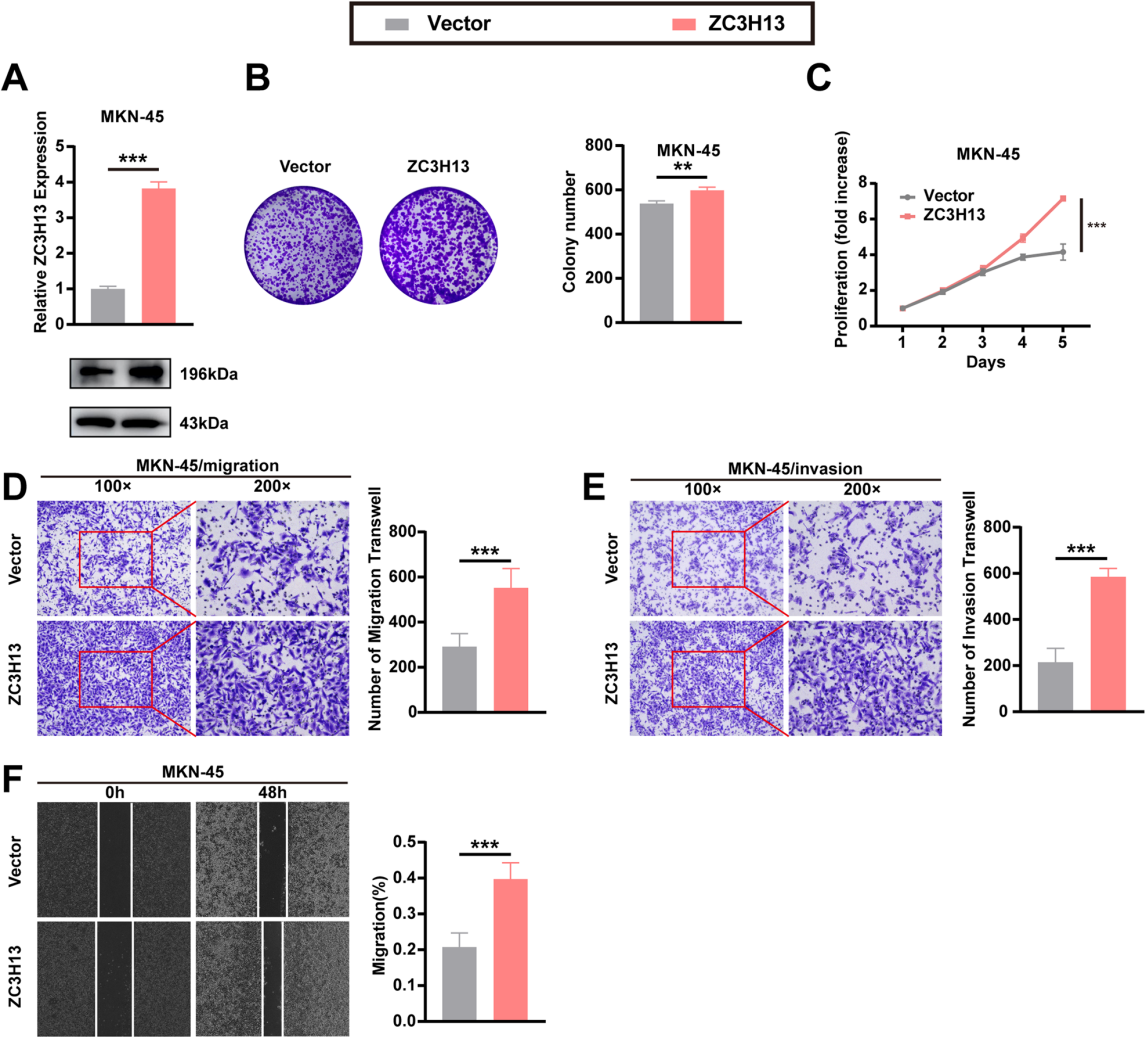
ZC3H13 overexpression promoted the proliferation, invasion, and migration of GC cells. **A** Increased mRNA and protein expression levels in MKN-45 cells after transfection with the overexpressing lentivirus. **B** ZC3H13 overexpression promoted the colony formation of GC cells. **C** ZC3H13 overexpression promoted the proliferation of GC cells. ZC3H13 overexpression promoted the migration (**D**) and invasion (**E**) of GC cells. **F** Wound healing assays revealed that ZC3H13 overexpression promoted GC cell migration. *P < 0.05, **P < 0.01, and ***P < 0.001 represent varying degrees of significance between the indicated groups.

The transfection of MKN-45 cells with the overexpression lentivirus increased the mRNA and protein expression of ZC3H13 (Fig. 3A). We found that ZC3H13 overexpression promoted the colony formation ability of MKN-45 cells, and CCK-8 assays demonstrated that ZC3H13 overexpression promoted the proliferative viability of MKN-45 cells (Fig. 3B, C). Transwell assays revealed that ZC3H13 overexpression significantly promoted the migration and invasion of MKN-45 cells (Fig. 3D, E), and wound healing assays also confirmed the promotional effect of ZC3H13 on the migration of MKN-45 cells (Fig. 3F).

### ZC3H13 overexpression did not affect on the function of normal gastric mucosal epithelial cells in vitro

We further established a ZC3H13 overexpression model using a lentivirus in GES-1 cells and performed cellular functionality experiments. Colony formation assays revealed that ZC3H13 overexpression did not affect the proliferation ability of GES-1 cells (Fig. S2A). Transwell and wound healing assays revealed that the migration and invasion ability of GES-1 cells was weak and was not affected by ZC3H13 overexpression (Fig. S2B–D).

### RNA-seq combined with multi-omics analysis identified possible effects of ZC3H13 on EMT

**Fig. 4:**
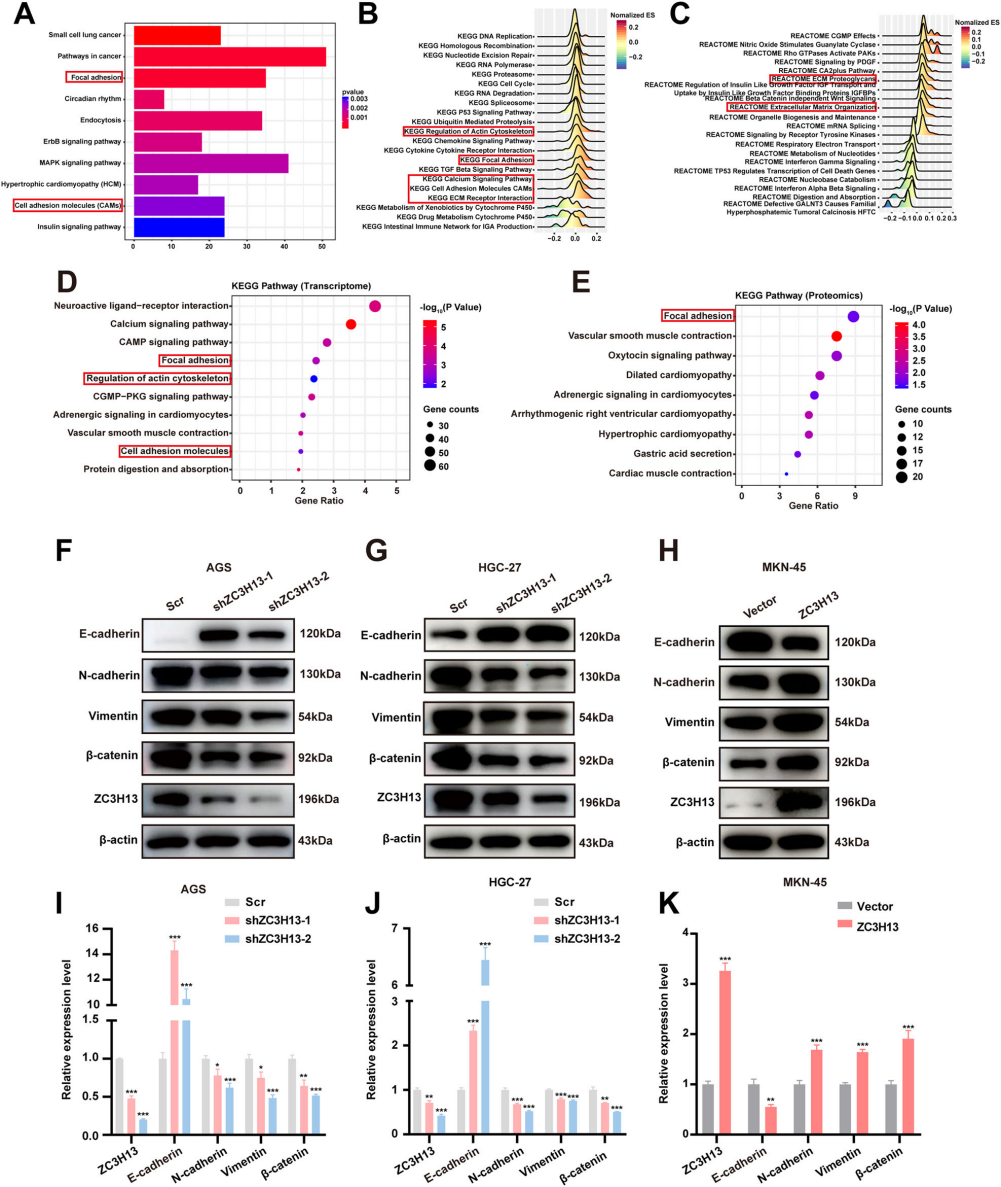
ZC3H13 may be involved in regulating the EMT signaling pathway. **A** Pathway enrichment analysis of differentially expressed genes in RNA-seq data. **B** GSEA of the KEGG dataset. **C** GSEA of the REACTOME dataset. **D** KEGG pathway enrichment for transcriptomics data. **E** KEGG pathway enrichment for proteomics data. Changes in the expression of EMT signaling pathway molecules after ZC3H13 was knocked down in AGS (**F**) and HGC-27 (**G**) cells. **H** Changes in the expression of EMT signaling pathway molecules after ZC3H13 was overexpressed in MKN-45 cells. **I**–**K** The band density was measured (ImageJ software) and normalized to that of β-actin.

To gain insight into the molecular mechanisms by which ZC3H13 promotes GC progression, we performed MeRIP-seq and RNA-seq using AGS cells with ZC3H13 knockdown, as well as multi-omics analysis of 33 GC tissues and corresponding normal paracarcinoma tissues. Pathway enrichment analysis of differential genes in the RNA-seq data revealed that ZC3H13 mainly regulates adhesion-related signaling pathways (Fig. 4A). Moreover, GSEA analyses based on KEGG and REACTOME datasets demonstrated the involvement of ZC3H13 in regulating focal adhesion, cell adhesion molecules (CAMs), and actin cytoskeleton (Fig. 4B, C). The analysis of the sequencing results of the 33 GC tissues was also consistent with the above, with KEGG pathway enrichment of the transcriptomic and proteomic data showing the involvement of ZC3H13 in regulating pathways such as those related to cell adhesion and the actin cytoskeleton (Fig. 4D, E). CAMs are a group of cell surface proteins that mediate intercellular adhesion [23]. The process of EMT is often accompanied by weakened intercellular adhesion and cytoskeletal remodeling [4, 24]. We therefore hypothesize that ZC3H13 is involved in regulating EMT. We subsequently found that knockdown of ZC3H13 increased E-cadherin expression and decreased N-cadherin, Vimentin, and β-catenin protein expression in AGS and HGC-27 cells (Fig. 4F, G), whereas the opposite results were found in MKN-45 cells overexpressing ZC3H13 (Fig. 4H). We also explored the effect of ZC3H13 overexpression on the expression of epithelial and mesenchymal markers in GES-1 cells. As shown in Fig. S3, ZC3H13 overexpression did not affect the expression of epithelial and mesenchymal markers in GES-1 cells. We demonstrated that ZC3H13 can promotes the progression of GC by promoting the process of EMT.

### MeRIP-seq and RNA-seq combined with multi-omics analysis identified SNTB1 as a downstream target of ZC3H13

**Fig. 5:**
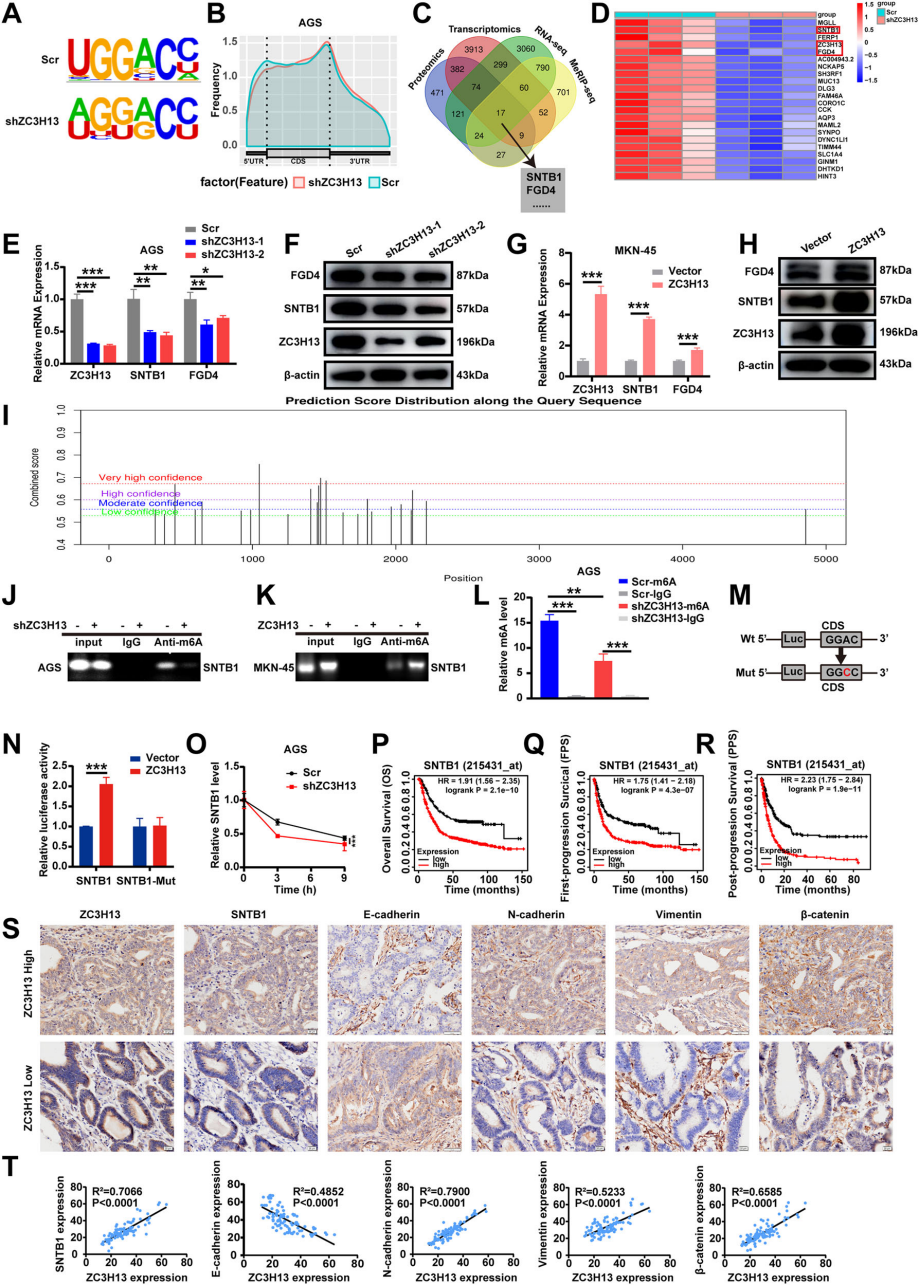
MeRIP-seq and RNA-seq combined with multi-omics analysis identified SNTB1 as the downstream target of ZC3H13. **A** Top sequence motif identified from MeRIP-seq peaks in control and ZC3H13-depleted cells. **B** Distribution of reduced m6A peaks generated by ZC3H13 inhibition across all the mRNAs. **C** Intersection of ZC3H13 related differential genes in MeRIP, RNA seq, and multi-omics data. **D** The top 20 genes with the greatest differences between the control and ZC3H13-depleted groups according to the RNA-seq data. Changes in the mRNA (**E**) and protein (**F**) expression levels of downstream target molecules after the knockdown of ZC3H13. Changes in the mRNA (**G**) and protein (**H**) expression levels of downstream target molecules after the overexpression of ZC3H13. **I** The predicted results for SNTB1 mRNA on the SRAMP website indicate potential sites for m6A modification. **J**, **K** MeRIP assays detected the m6A modification of SNTB1 by using anti-IgG and anti-m6A antibodies. **L** RNA obtained after MeRIP was reverse transcribed for RT-qPCR to detect the m6A modification level of SNTB1. **M** The wild-type or mutant m6A consensus sequence is linked to firefly luciferase. **N** Effect of ZC3H13 overexpression on the luciferase activity of the wild-type and mutant SNTB1 fusion reporter genes. **O** Expression of SNTB1 mRNA in control and shZC3H13 AGS cells treated with actinomycin D (5 μg/ml) for different durations. Relationship between the expression of SNTB1 and OS (**P**), FPS (**Q**), and PPS (**R**) in GC tissue. **S** Representative images showing high or low expression of ZC3H13, SNTB1, E-cadherin, N-cadherin, Vimentin, and β-catenin in 60 gastric tumor samples (magnification, 200×). **T** Correlations of ZC3H13 expression with SNTB1, E-cadherin, N-cadherin, Vimentin and β-catenin expression in 60 gastric tumor tissues. *P < 0.05, **P < 0.01, and ***P < 0.001 represent varying degrees of significance between the indicated groups.

To gain insight into the mechanism by which ZC3H13 promotes the malignant progression of cancer, we further analyzed the results from MeRIP-seq, RNA-seq, and multi-omics analyses. The MeRIP-seq results revealed that “GGAC” was the major common motif in the negative control and ZC3H13 knockdown samples (Fig. 5A), and m6A peaks were abundant in the coding sequences (CDS), especially near the stop codon (Fig. 5B), which is consistent with published reports [25]. We then took the intersection of differentially expressed genes from the MeRIP-seq, RNA-seq, and tissue transcriptomic and proteomic results, and screened 17 candidate genes (Fig. 5C). Since the methyltransferase ZC3H13 positively mediates m6A modification, we focused on genes with reduced m6A peaks, and combined with the list of genes with large differences in the RNA-seq data (Fig. 5D), we ultimately identified SNTB1 and FGD4 as potential downstream target genes for ZC3H13. Both the mRNA and protein expression of the candidate downstream target genes were significantly reduced after the knockdown of ZC3H13, whereas the opposite effect occurred after the overexpression of ZC3H13 (Fig. 5E–H). We then selected SNTB1, which had the most pronounced differences, as a possible downstream target gene for ZC3H13.

To identify specific m6A sites modified by ZC3H13, the mRNA sequence of SNTB1 was predicted using the SRAMP website (http://www.cuilab.cn/sramp/). The predictions revealed three potential m6A modifications sites with very high confidence in the mRNA sequence of SNTB1 (Fig. 5I). MeRIP experiments with specific primers designed according to the possible m6A modification sites in the CDS region of SNTB1 demonstrated that the knockdown of ZC3H13 reduced the m6A modification of SNTB1 (Fig. 5J) and that the overexpression of ZC3H13 increased the m6A modification of SNTB1 (Fig. 5K). In addition, we further demonstrated quantitatively via MeRIP-qPCR that the m6A modification level of SNTB1 was reduced after the knockdown of ZC3H13 (Fig. 5L). We then constructed luciferase reporter systems containing wild-type or mutant SNTB1 to demonstrate the effect of m6A modification on SNTB1 expression. The adenylate in the m6A common sequence (RRACH) of mutant SNTB1 was replaced by cytosine using a point mutation kit (Fig. 5M). Luciferase reporter assays revealed that the transcriptional level of wild-type SNTB1, but not the SNTB1 mutation, significantly increased with ZC3H13 overexpression (Fig. 5N). In addition, we examined the decay rate of SNTB1 mRNA in AGS cells with ZC3H13 knockdown and their corresponding negative control cells. SNTB1 mRNA expression was reduced by the addition of actinomycin D, and the half-life of SNTB1 mRNA was significantly shorter when ZC3H13 was knocked down compared to the negative control cells (Fig. 5O). Furthermore, survival analysis revealed that patients with high SNTB1 expression had significantly lower OS, FPS and PPS (Fig. 5P–R). We next performed IHC staining using tissue microarrays from the 60 GC patients mentioned above, which revealed that the protein expression of ZC3H13 in the GC tissues was significantly and positively correlated with SNTB1, as well as with the EMT-associated molecules N-cadherin, Vimentin and β-catenin, and significantly negatively correlated with E-cadherin (Fig. 5S, T). These results suggest that ZC3H13 increases the expression of SNTB1 by way of m6A modification and further promotes the malignant progression of GC through the EMT signaling pathway.

### YTHDF1 enhanced SNTB1 mRNA stability in a m6A-dependent manner

**Fig. 6:**
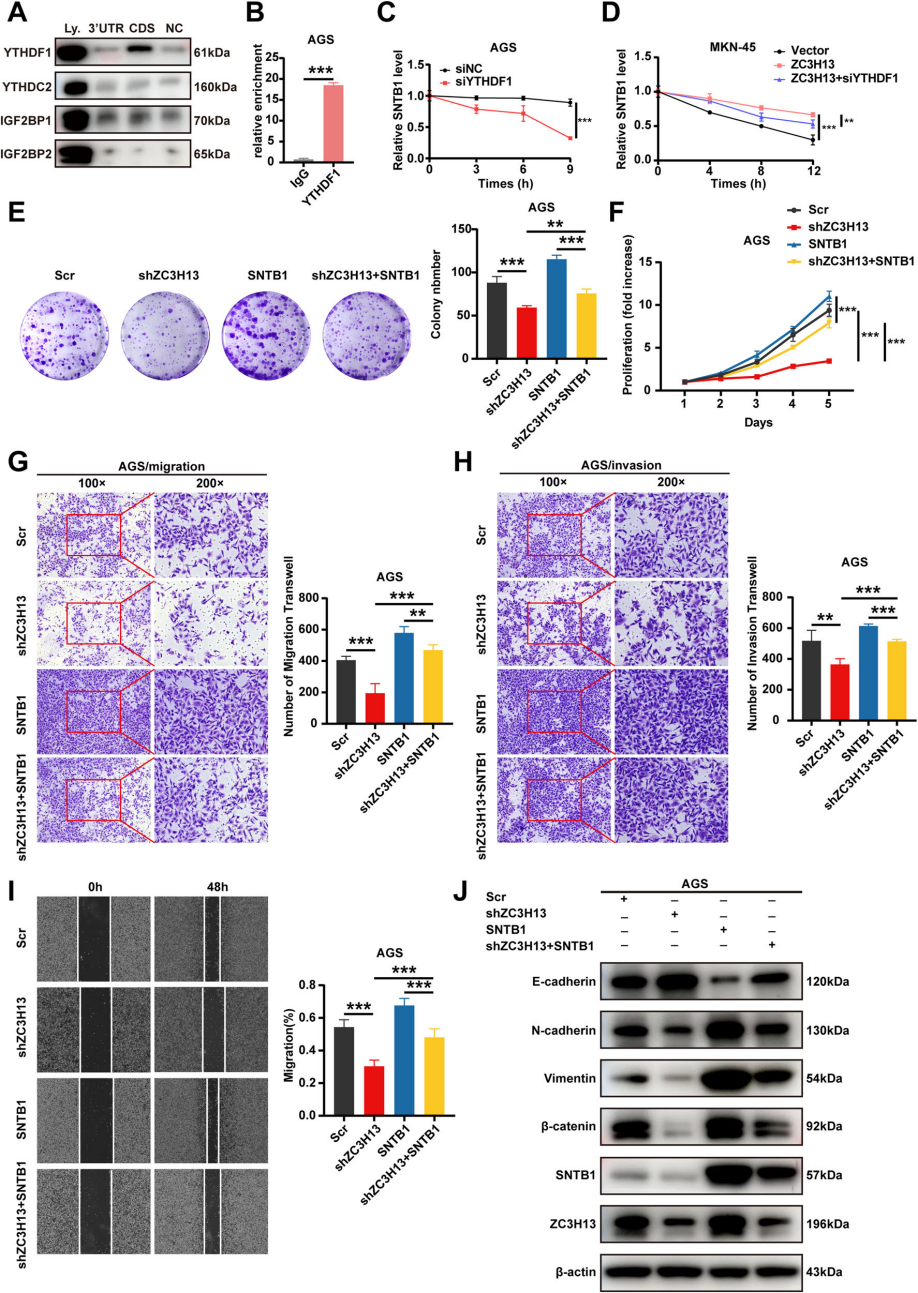
YTHDF1 enhanced SNTB1 mRNA stability in a m6A-dependent manner, and SNTB1 overexpression prevented the inhibition of GC cell proliferation, migration, and invasion caused by the downregulation of ZC3H13. **A** Western blotting after an RNA pull-down assay with cell lysate (Ly.), SNTB1 CDS and 3’UTR regions, and beads only (NC) in AGS cells. **B** qRT-PCR analysis of the RIP assay results in AGS cells revealed direct binding between the YTHDF1 protein and SNTB1 mRNA. **C** The mRNA attenuation rate of SNTB1 in AGS cells treated with actinomycin D (5 μg/ml) after YTHDF1 inhibition. **D** Effect of YTHDF1 knockdown on the SNTB1 RNA decay rate in AGS cells overexpressing ZC3H13. **E** Effect on colony formation in AGS cells after the overexpression of SNTB1. **F** Effect on the proliferative viability of AGS cells after the overexpression of SNTB1. Effect of SNTB1 overexpression on migration **G** and invasion **H** in AGS cells. **I** Wound healing assay showing the effect of SNTB1 overexpression on the migration ability of AGS cells. **J** Effect of SNTB1 overexpression on the protein expression of EMT signaling pathway molecules. *P < 0.05, **P < 0.01, and ***P < 0.001 represent varying degrees of significance between the indicated groups.

Previous studies have identified some m6A readers, such as those in the TYH family and the IGF2BP family [12, 26, 27], which play important roles in m6A regulation. To elucidate the specific m6A readers of SNTB1, we performed a streptavidin RNA pull-down assay to screen for SNTB1-associated m6A readers. Interestingly, YTHDF1 (but not other members of the IGF2BP family or the YTH family) specifically bound the CDS region of SNTB1 in AGS cells (Fig. 6A). RIP analysis also verified the existence of a direct interaction between YTHDF1 and SNTB1 mRNA in AGS cells (Fig. 6B). Consistent with our hypothesis, both SNTB1 protein and mRNA expression levels were significantly reduced after the YTHDF1 expression in AGS cells was inhibited by siRNA (Fig. S4A, B). The stability of SNTB1 mRNA in AGS cells was significantly decreased and the half-life was significantly shortened after YTHDF1 inhibition (Fig. 6C). Furthermore, the knockdown of YTHDF1 restored the effect of high ZC3H13 expression on SNTB1 expression (Fig. S4C) and the increase in SNTB1 mRNA stability caused by the overexpression of ZC3H13 was partially restored after knockdown of YTHDF1 (Fig. 6D). Taken together, our findings suggest that SNTB1 transcripts are recognized by the m6A reader YTHDF1, which maintains the stability of its mRNA to prevent its degradation and increases its expression through a m6A-YTHDF1-dependent mechanism.

### ZC3H13 promotes the malignant progression of GC by upregulating SNTB1

We next constructed SNTB1-overexpressing lentiviruses and SNTB1-knockdown siRNAs to elucidate whether ZC3H13 promotes GC progression by increasing SNTB1 expression. The overexpression of SNTB1 increased AGS cell proliferation and rescued the attenuated proliferation caused by the knockdown of ZC3H13 (Fig. 6E, F). The overexpression of SNTB1 also increased the migration and invasion of AGS cells and rescued the above cellular phenotypes caused by the knockdown of ZC3H13 (Fig. 6G–I). In addition, we found that the overexpression of SNTB1 significantly reduced E-cadherin expression and restored the elevated E-cadherin expression caused by the knockdown of ZC3H13 (Fig. 6J). Moreover, SNTB1 overexpression increased the expression of the mesenchymal markers N-cadherin, Vimentin, and β-catenin and restored the reduced expression caused by the knockdown of ZC3H13 (Fig. 6J).

**Fig. 7:**
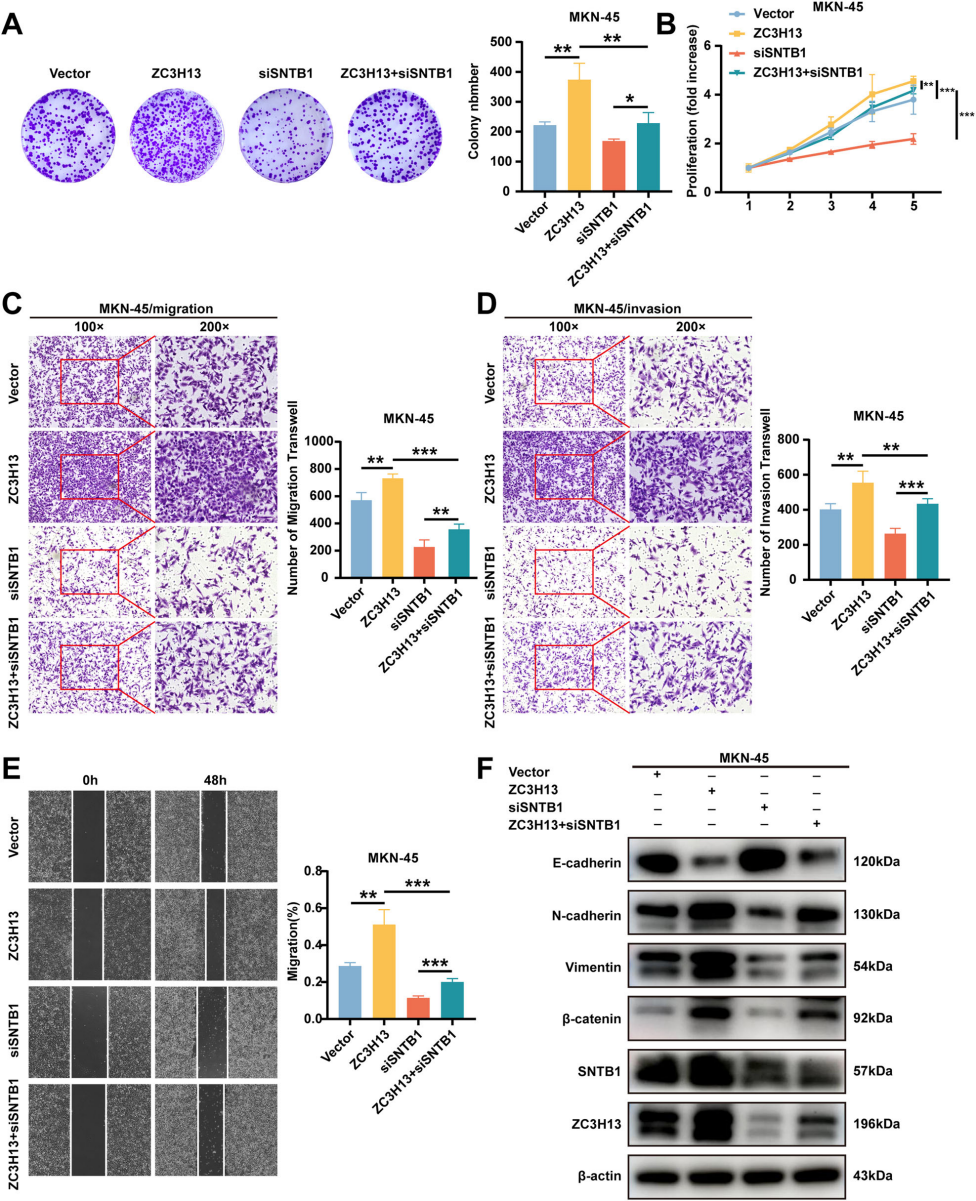
The knocking down of SNTB1 inhibited the promotion of GC cell proliferation, migration, and invasion caused by ZC3H13 overexpression. **A** Effect of SNTB1 knockdown on colony formation in MKN-45 cells. **B** Effect on proliferative viability in MKN-45 cells after knockdown of SNTB1. Effect of SNTB1 knockdown on migration (**C**) and invasion (**D**) in MKN-45 cells. **E** Wound healing assay showing the effect of SNTB1 knockdown on the migration ability of AGS cells. **F** Effect of SNTB1 knockdown on the protein expression of EMT signaling pathway molecules. *P < 0.05, **P < 0.01, and ***P < 0.001 represent varying degrees of significance between the indicated groups.

Conversely, SNTB1 knockdown inhibited the proliferation, migration and invasion of MKN-45 cells and reversed the effects of ZC3H13 overexpression on the above phenotypes (Fig. 7A–E). In addition, reduced SNTB1 expression inhibited EMT and reversed the promoting effect of ZC3H13 overexpression on EMT (Fig. 7F). These results indicate that ZC3H13 promotes the progression of GC by upregulating the expression of SNTB1.

### ZC3H13 promoted the proliferation and metastasis of GC cells in vivo

**Fig. 8:**
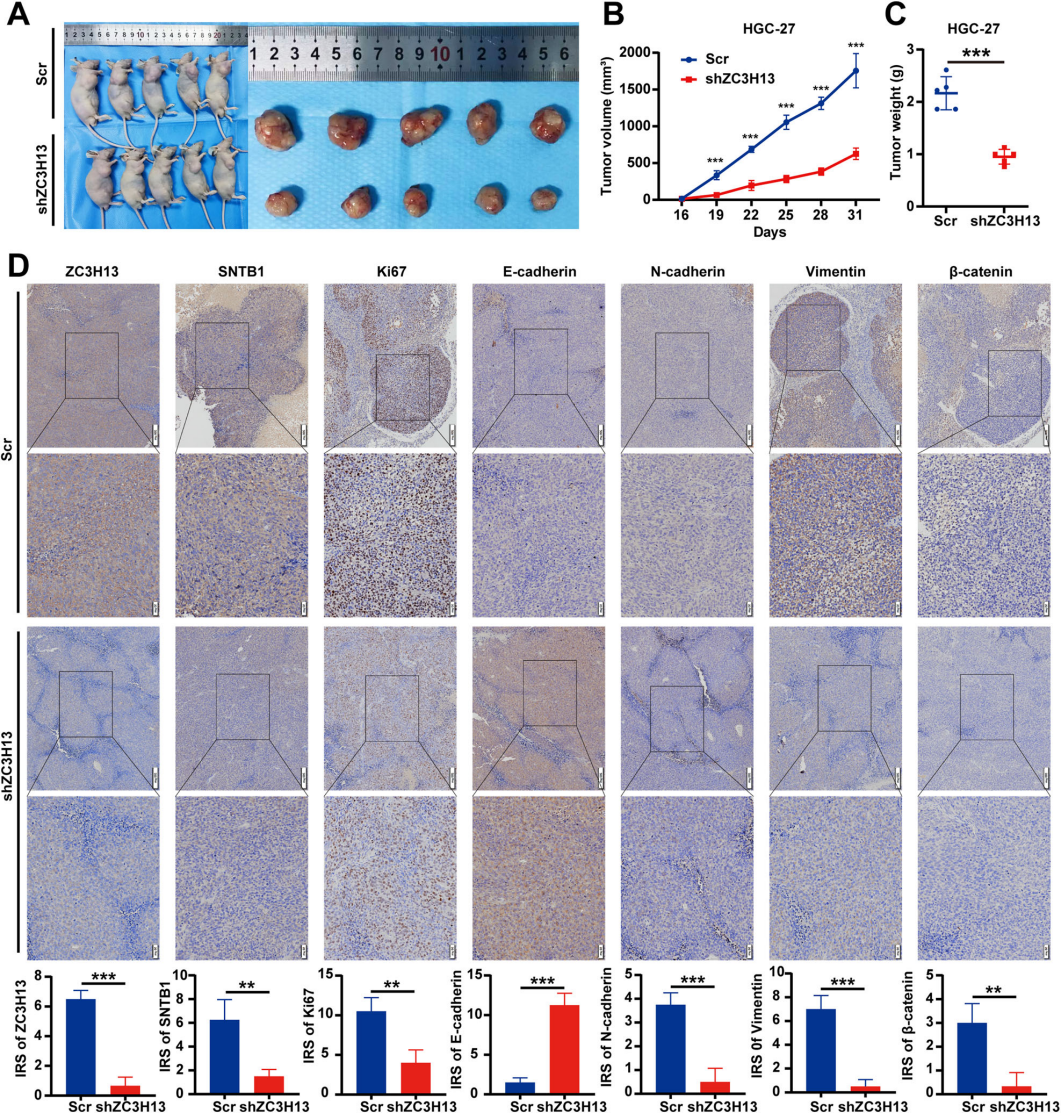
ZC3H13 knockdown inhibited the proliferation, migration, and invasion of GC cells in vivo. **A** We established a xenograft model by injecting HGC-27 cells pretransfected with shZC3H13. Tumor diameter was measured every 3 days, and the tumor volume (**B**) and weight (**C**) were determined. **D** IHC staining of ZC3H13, SNTB1, Ki67, and EMT signaling pathway molecules in xenograft tumor sections (magnification, 40× and 100×). *P < 0.05, **P < 0.01, and ***P < 0.001 represent varying degrees of significance between the indicated groups.

To elucidate the oncogenic role of ZC3H13 in GC, we performed in vivo experiments and established a subcutaneous tumor model. Tumor volume and weight were lower in the ZC3H13-knockdown group than in the control group (Fig. 8A–C). We performed IHC experiments and found that the knockdown of ZC3H13 resulted in a significant reduction of SNTB1 and Ki67 expression levels in the xenograft tumors compared to the controls (Fig. 8D). In addition, our IHC staining for EMT markers further demonstrated the inhibitory effect of ZC3H13 knockdown on EMT (Fig. 8D).

**Fig. 9:**
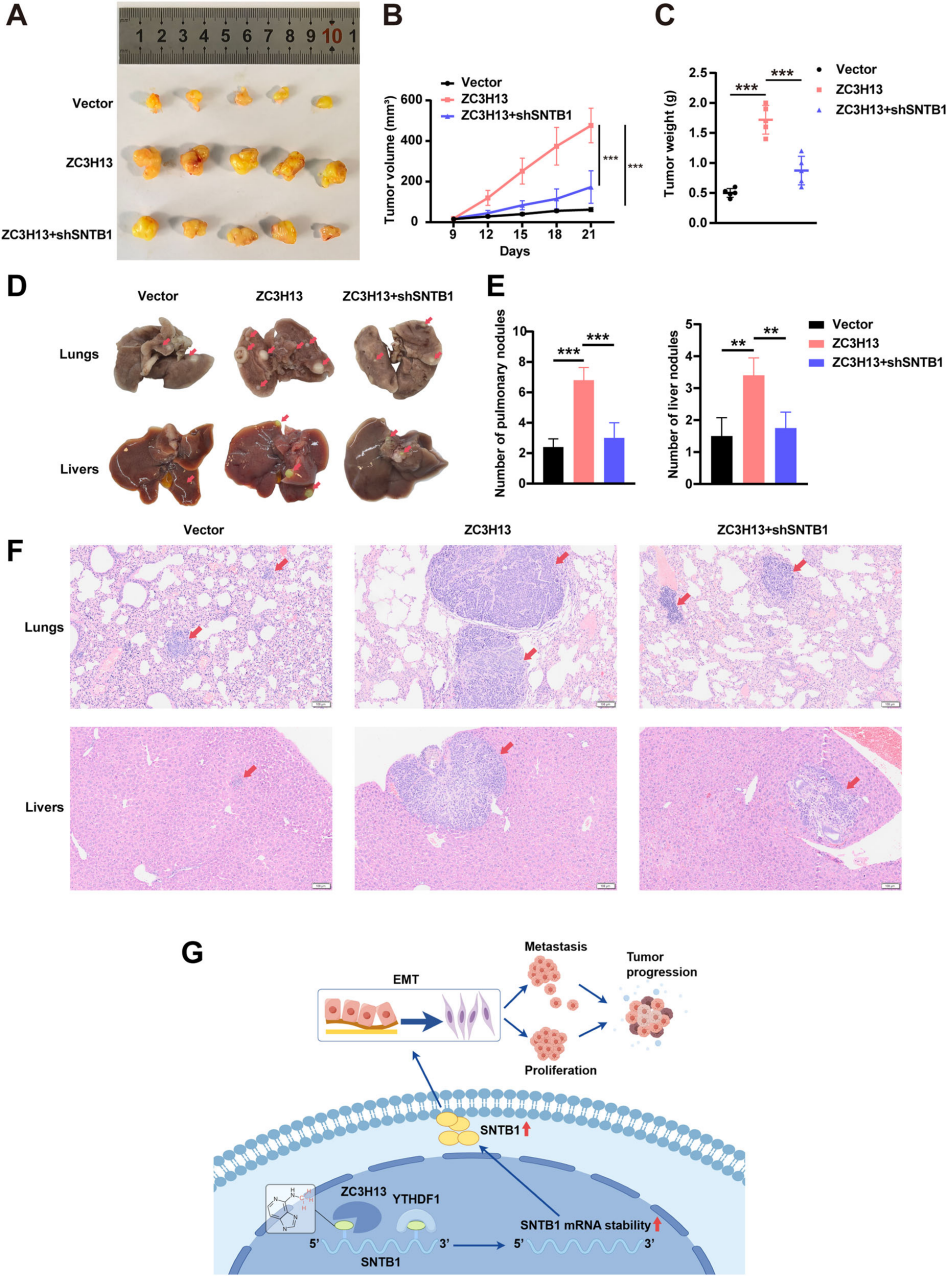
SNTB1 knockdown restored the promotion of GC metastasis in vivo by ZC3H13 overexpression. **A** Effects of ZC3H13 overexpression and SNTB1 knockdown on GC cells proliferation in vivo. Tumor diameter was measured every 3 days, and the tumor volume (**B**) and weight (**C**) were determined. **D** Representative images of the effects of ZC3H13 overexpression and SNTB1 knockdown on lung and liver metastasis of GC cells. **E** Quantitative results of the number of nodules in lung and liver metastases. **F** H&E staining of lung and liver metastases (magnification, 40×). **G** The schematic diagram of the mechanism by which ZC3H13 promotes GC progression in this study. *P < 0.05, **P < 0.01, and ***P < 0.001 represent varying degrees of significance between the indicated groups.

To further demonstrate that ZC3H13 promotes the progression of GC by regulating SNTB1, we investigated the effects of ZC3H13 overexpression and SNTB1 knockdown on proliferation and metastasis in vivo. The results indicated that ZC3H13 overexpression promoted the proliferation of GC in vivo, with tumor volume and weight significantly higher than that of the control group, but these effects were reversed by SNTB1 knockdown (Fig. 9A–C). In addition, we established the lung and liver metastasis models of GC by injecting MKN-45 cells via tail vein and spleen. The results indicated that ZC3H13 overexpression promoted the metastasis of GC, the number of metastatic foci in the lungs and livers of the mice were more numerous and larger in size compared with those of the control group, but these effects were reversed by SNTB1 knockdown (Fig. 9D–F).

Based on the above experiments, we conclude that ZC3H13 regulates the expression of SNTB1 in a m6A-YTHDF1-dependent manner and promotes the malignant progression of GC through the EMT signaling pathway (Fig. 9G).

## Discussion

Distant metastasis is one of the most prominent features of malignant tumors and is the cause of death in 90% of cancer patients [28]. The acquisition of invasive and stem cell-like properties by cancer cells is a prerequisite for cancer metastasis [29], and it is by influencing the EMT process that cancer cells achieve this condition [30, 31]. EMT is an important factor in the initiation, invasion, and metastasis of cancer cells and is a major cause of early dissemination in GC [32]. The pathogenesis by which m6A dysregulation promotes tumor progression has remained a hot research topic in recent years. m6A regulators include writers (METTL3, METTL14, KIAA1429, and ZC3H13), erasers (ALKBH5 and FTO) and readers (YTHDF1/2/3, IGF2BP1/2/3), which are thought to play important roles in regulating cancer progression. Indeed, m6A writers (e.g., METTL3, METTL14, and KIAA1429) affect the malignant progression of GC through different mechanisms [14, 33–36]. In particular, m6A regulators are involved in regulating cancer metastasis. For example, ALKBH5 is downregulated in gastric cancer and inhibits gastric cancer metastasis by inhibiting WRAP53 translation in a m6A-dependent manner [37]. YTHDF3 binds to and degrades ZFP41 mRNA, inhibiting the Snail and EMT pathways and thus suppressing hepatocellular carcinoma metastasis [38]. However, the role and molecular mechanism of ZC3H13 in the occurrence and development of GC have not yet been reported, especially its involvement in regulating EMT and GC metastasis, which is still largely unclear.

ZC3H13 is a component of the m6A methylation complex and interacts with WTAP, virilizer and RBM15 to regulate the level of m6A modification of mRNA [13, 39, 40]. Recent reports have highlighted the controversial role of ZC3H13 in cancer progression. For example, ZC3H13 promotes stemness and chemotherapy resistance in cervical cancer cells [16]. In contrast, some studies suggest that ZC3H13 is a tumor suppressor gene for colorectal cancer and breast cancer [17, 21]. In this study, we reported for the first time that increased expression of ZC3H13 leads to poor prognosis in patients with GC. Our study identifies ZC3H13 as a critical facilitator of GC metastasis, driving cell proliferation, invasion, migration, and the EMT process in GC cells. Intriguingly, its overexpression did not induce proliferation or a metastatic phenotype in the normal gastric mucosal epithelial cell line GES-1, nor did it alter the expression of epithelial and mesenchymal markers. This discrepancy may be attributed to the distinct signaling environments between cancer cells and normal cells. The unique microenvironment of cancer cells likely enables ZC3H13 to activate the EMT process and disrupt epithelial integrity. From a translational perspective, the cancer-promoting role of ZC3H13 highlights its potential as a therapeutic target for GC, thereby reducing collateral damage to healthy tissues. In summary, we have demonstrated that ZC3H13 regulates EMT and promotes the malignant phenotype of gastric cancer.

As a type of methyltransferase, ZC3H13 often functions by regulating the m6A modification of downstream molecules [16]. We used MeRIP-seq and RNA-seq combined with multi-omics analysis to screen SNTB1 as a downstream target of ZC3H13, and demonstrated that SNTB1 was regulated by ZC3H13 in a m6A-modified manner and that ZC3H13 promoted the EMT process in GC cells, which was closely related to SNTB1 expression. SNTB1 is a member of the syntrophin family. Syntrophins ensure that signaling proteins can localize to specific membrane structural domains and appropriately regulate signaling pathways; at the same time, syntrophins bind to cytoskeletal proteins and lead to various cellular responses by regulating the cytoskeleton [41]. SNTB1 has been reported in the field of cancer as a promoter of colorectal cancer progression through activation of the ERK and AKT signaling pathways [42]. Another study reported that SNTB1 promotes the malignant progression of colorectal cancer through the YAP1 and WNT/β-catenin pathways [43]. However, the reason for the up-regulation of SNTB1 expression in GC has not been reported. We found that SNTB1 expression was modulated by ZC3H13-associated m6A modification in the CDS of SNTB1 mRNA, which upregulated the expression of SNTB1 through m6A modification, thereby positively regulating the EMT process to promote the proliferation, invasion, and migration of GC cells.

According to previous reports, m6A readers are involved in the control of mRNA fate, and both the YTH family and the IGF2BP family are associated with the stability of methylated mRNAs [44, 45]. Different reader proteins respond differently to m6A modification, thereby mediating different roles of target genes. For example, YTHDF1 recognizes FTO-mediated m6A modification of ANGPTL4 and promotes translational enhancement of ANGPTL4 mRNA, which promotes breast cancer metastasis [46]; METTL3-mediated m6A modification stabilizes PAK6 mRNA through IGF2BP1 recognition and promotes cervical cancer growth and metastasis [47]. We verified the modification of SNTB1 mRNA by ZC3H13 by MeRIP and luciferase reporter gene assays, and that ZC3H13 is dependent on the m6A reader YTHDF1 for its m6A-modifying effects. RNA pull-down and RIP assays confirmed that YTHDF1 can directly bind to the m6A site in the CDS region of SNTB1 and control the stability of SNTB1 mRNA in a m6A-dependent manner. In summary, we elucidated the high expression of ZC3H13 in GC tissue and revealed its molecular mechanism of promoting GC progression, indicating its potential role in predicting GC metastasis.

## Conclusions

In conclusion, our study is the first to elucidate the oncogenic role of the ZC3H13-mediated m6A mechanism in GC. High expression of ZC3H13 increases the m6A modification level of SNTB1 and increases its expression in a YTHDF1-dependent manner, thereby promoting the proliferation, invasion, and migration ability of GC cells by actively regulating the EMT process. Our findings highlight the functional value of m6A methylation as tumor markers and contribute to the search for potential prognostic markers and therapeutic targets for GC.

## Supplementary information

Figure S1

Figure S2

Figure S3

Figure S4

Supplementary Tables

Supplementary Figures

Original western blot

## Data Availability

The datasets used and/or analyzed during the current study are available from the corresponding author upon reasonable request.
