# Supplementary figures and images for: ZC3H13 mediates N6-methyladenosine modification of SNTB1 to promote epithelial-mesenchymal transition in gastric cancer
Source: Cell Death Dis. 2025 Aug 7;16(1):596. doi: 10.1038/s41419-025-07889-2 (PMC12331926; doi:10.1038/s41419-025-07889-2)

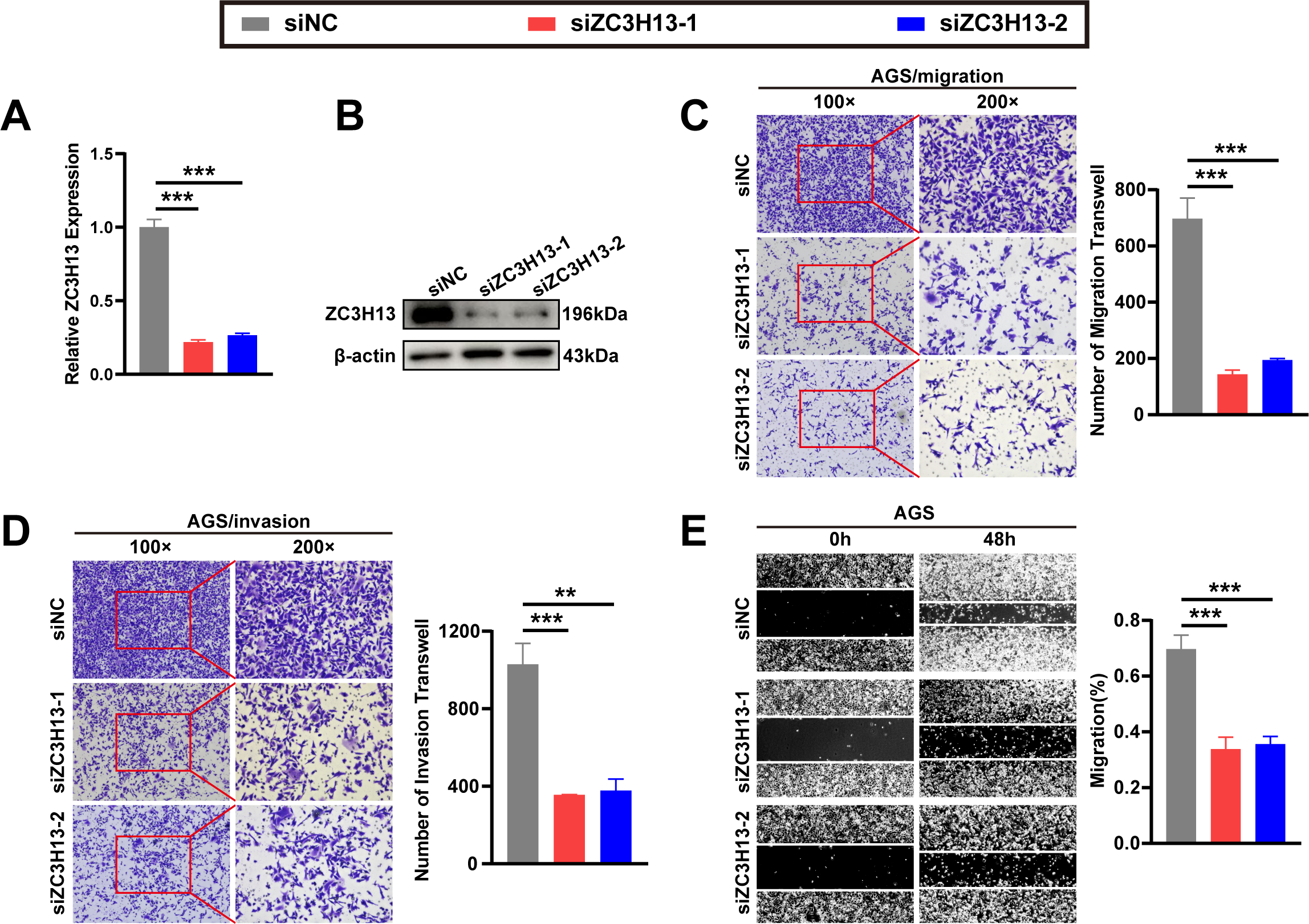

Supplement: Supplementary file 1 — Figure S1 [file 41419_2025_7889_MOESM1_ESM.tif]

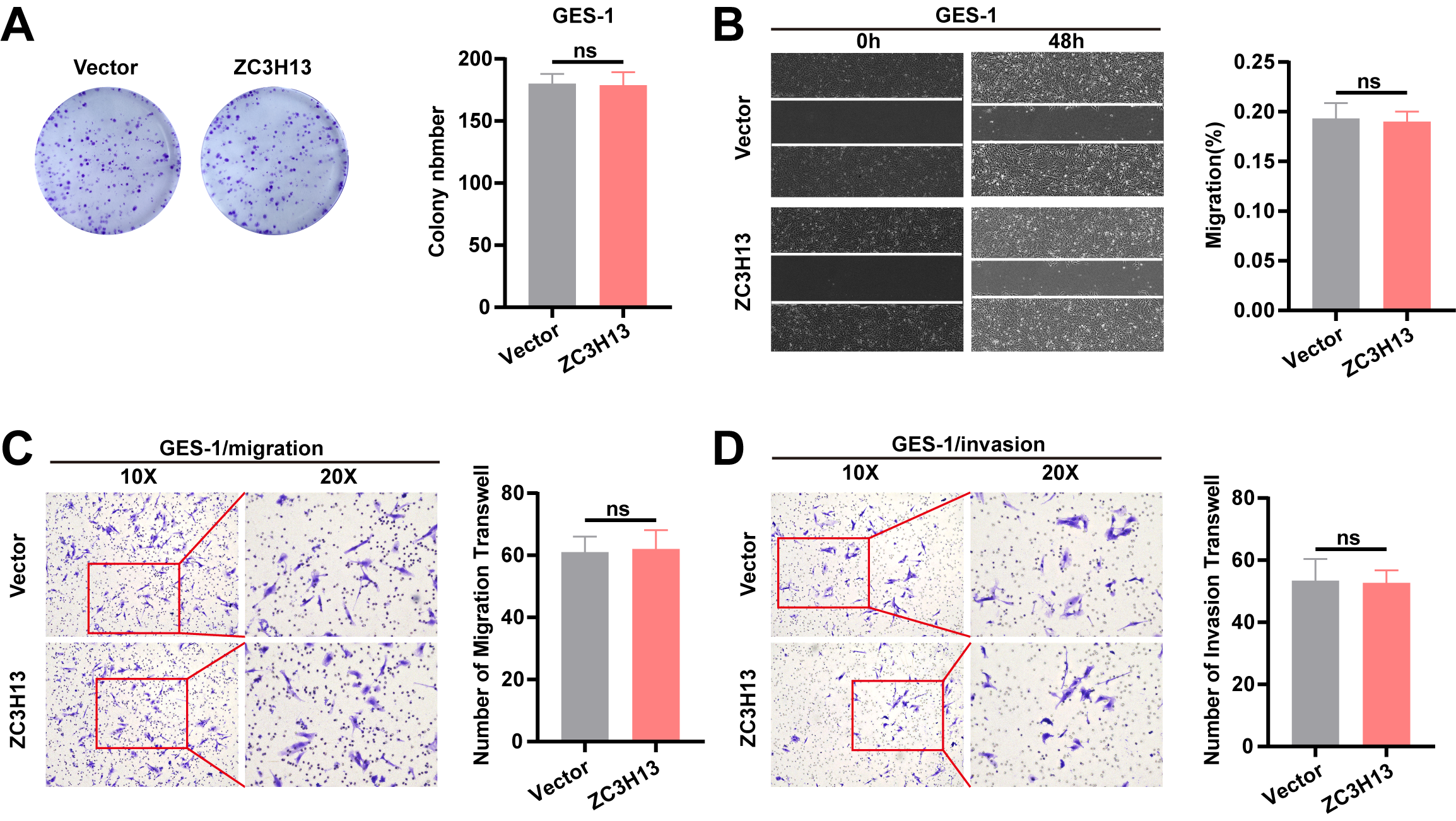

Supplement: Supplementary file 2 — Figure S2 [file 41419_2025_7889_MOESM2_ESM.tif]

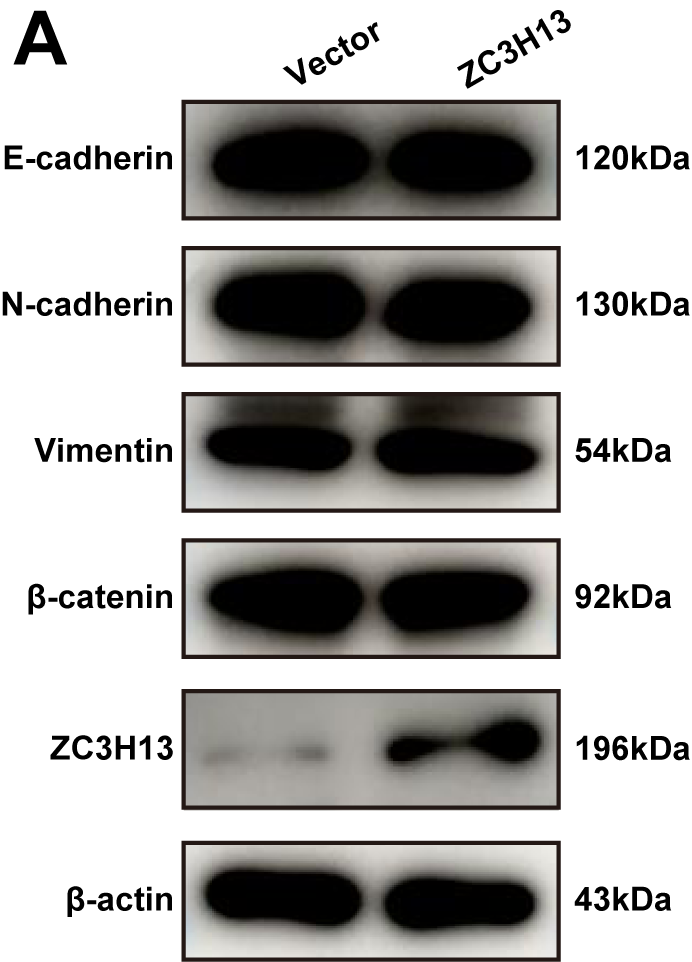

Supplement: Supplementary file 3 — Figure S3 [file 41419_2025_7889_MOESM3_ESM.tif]

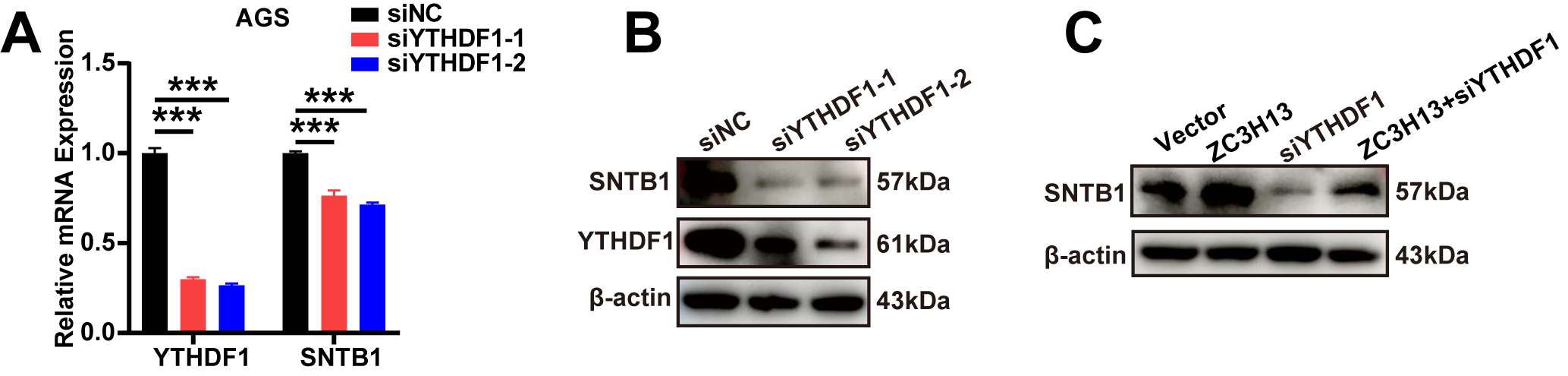

Supplement: Supplementary file 4 — Figure S4 [file 41419_2025_7889_MOESM4_ESM.tif]
