# Supplementary material for: ZC3H13 mediates N6-methyladenosine modification of SNTB1 to promote epithelial-mesenchymal transition in gastric cancer
Source: Cell Death Dis. 2025 Aug 7;16(1):596. doi: 10.1038/s41419-025-07889-2 (PMC12331926; doi:10.1038/s41419-025-07889-2)
Supplement: Supplementary file 5 — Supplementary Tables [file 41419_2025_7889_MOESM5_ESM.docx]

**Supplementary Table 1.** Primers used in this study for qRT-PCR analysis

| Primer names | Forward (5’-3’) | Reverse (5’-3’) |
| --- | --- | --- |
| ZC3H13 | CCCAGAGAAAGCAGAGTCCT | CATGGCGTCGATCATGAGAC |
| SNTB1 | GCTGCTGGAAGTGAAGTA | GTGGAAGGAGAAGGACTG |
| FGD4 | GACAACCACACCTCAACA | TCCTCCTCACATTCCATCT |
| YTHDF1 | GACGACATCCACCGCTCCATTAAG | CCCACTCCCATTGACGCTGAAG |
| GAPDH | TGCACCACCAACTGCTTAGC | GGCATGGACTGTGGTCATGAG |

**Supplementary Table 2.** Primary antibodies applied in this study

| Antigens | Catalog | Manufacturer | Application |
| --- | --- | --- | --- |
| β-actin | 20536-1-AP | Proteintech | 1:5000 for WB |
| ZC3H13 | HPA040140 | Sigma | 1:1000 for WB and 1:500 for IHC |
| SNTB1 | GTX132898 | GeneTex | 1:1000 for WB and 1:500 for IHC |
| E-cadherin | 20874-1-AP | Proteintech | 1:5000 for WB and 1:2000 for IHC |
| N-cadherin | 66219-1-Ig | Proteintech | 1:5000 for WB and 1:2000 for IHC |
| Vimentin | 60330-1-Ig | Proteintech | 1:5000 for WB and 1:2000 for IHC |
| β-catenin | 51067-2-AP | Proteintech | 1:5000 for WB and 1:1000 for IHC |
| FGD4 | GTX109859 | GeneTex | 1:1000 for WB |
| IGF2BP1 | 22803-1-AP | Proteintech | 1:5000 for WB |
| IGF2BP2 | 11601-1-AP | Proteintech | 1:2000 for WB |
| YTHDC2 | 27779-1-AP | Proteintech | 1:1000 for WB |
| YTHDF1 | db14438 | Diagbio | 1:1000 for WB |
