# Supplementary material for: ZC3H13 mediates N6-methyladenosine modification of SNTB1 to promote epithelial-mesenchymal transition in gastric cancer
Source: Cell Death Dis. 2025 Aug 7;16(1):596. doi: 10.1038/s41419-025-07889-2 (PMC12331926; doi:10.1038/s41419-025-07889-2)
Supplement: Supplementary file 6 — Supplementary Figures [file 41419_2025_7889_MOESM6_ESM.docx]

**Supplementary Figures**

**Figure S1.** ZC3H13 mRNA (A) and protein (B) expression decreased in AGS cells after transfection with either siZC3H13-1 or siZC3H13-2. ZC3H13 knockdown inhibited the migration (C) and invasion (D) ability of AGS cells. (E) Wound healing assays revealed that ZC3H13 knockdown weakened the migration ability of AGS cells.

**Figure S2.** (A) Effect of ZC3H13 overexpression on the colony formation of GES-1 cells. (B) Wound healing assays demonstrated the effect of ZC3H13 overexpression on the migration ability of GES-1 cells. Transwell assays demonstrated the effects of ZC3H13 overexpression on the migration (C) and invasion (D) ability of GES-1 cells.

**Figure S3.** (A) ZC3H13 overexpression did not affect the expression of epithelial and mesenchymal markers in GES-1 cells.

**Figure S4.** Inhibition of YTHDF1 expression reduced the mRNA (A) and protein (B) expression of SNTB1 in AGS cells. (C) Promotion of SNTB1 expression by ZC3H13 overexpression in MKN-45 cells was reversed by YTHDF1 knockdown.
