# Supplementary material for: ZC3H13 mediates N6-methyladenosine modification of SNTB1 to promote epithelial-mesenchymal transition in gastric cancer
Source: Cell Death Dis. 2025 Aug 7;16(1):596. doi: 10.1038/s41419-025-07889-2 (PMC12331926; doi:10.1038/s41419-025-07889-2)

Figure 2B

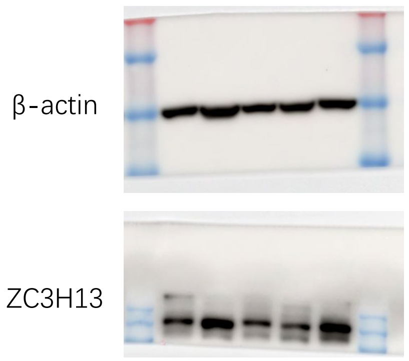

Figure 2C

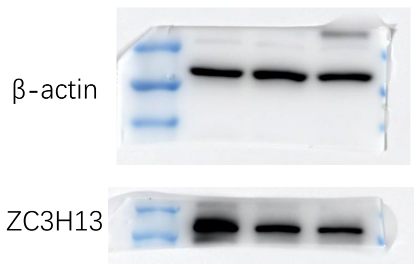

Figure 2D

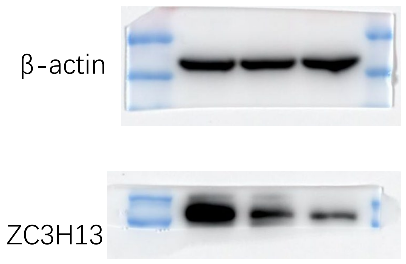

Figure 3A

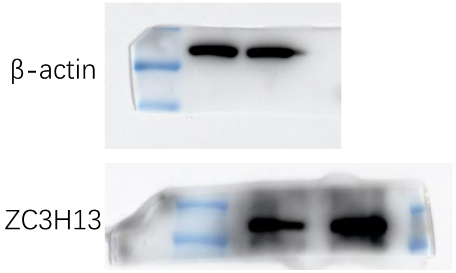

Figure 4F

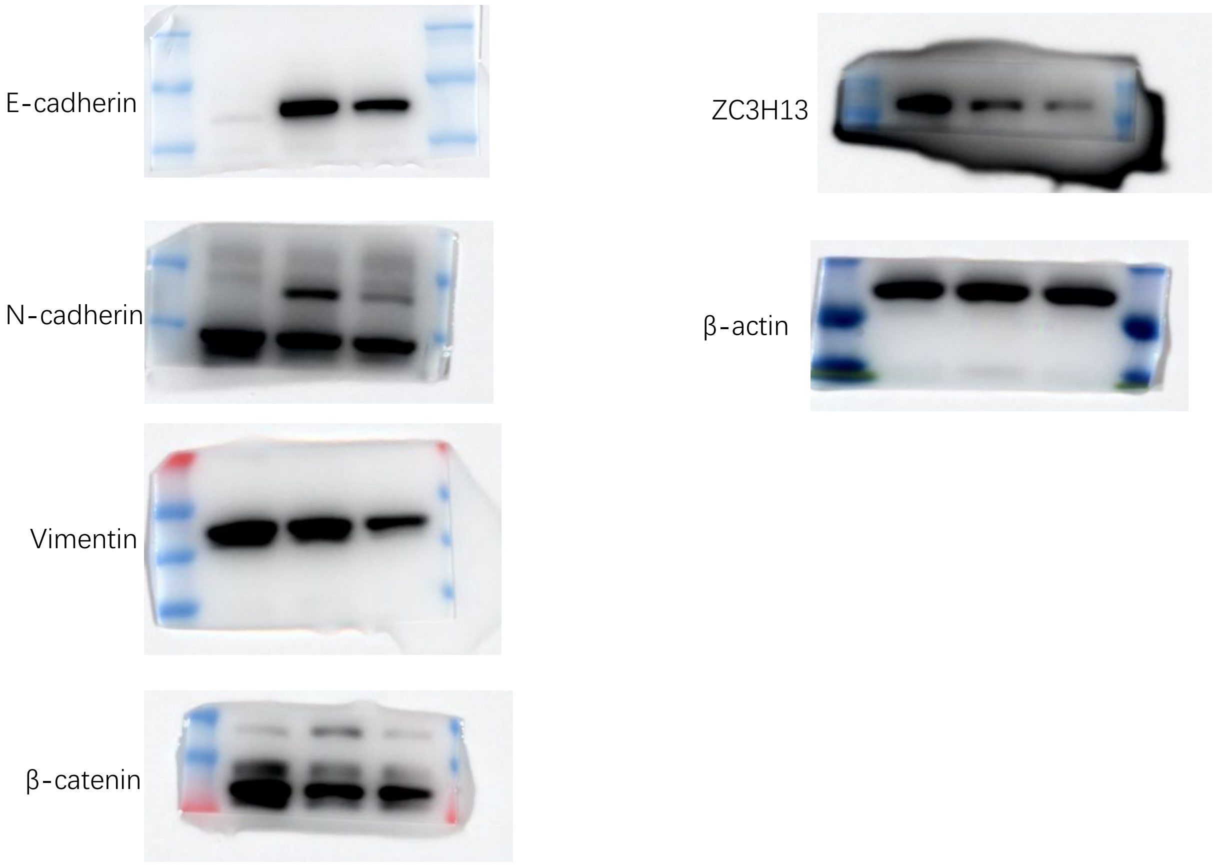

Figure 4G

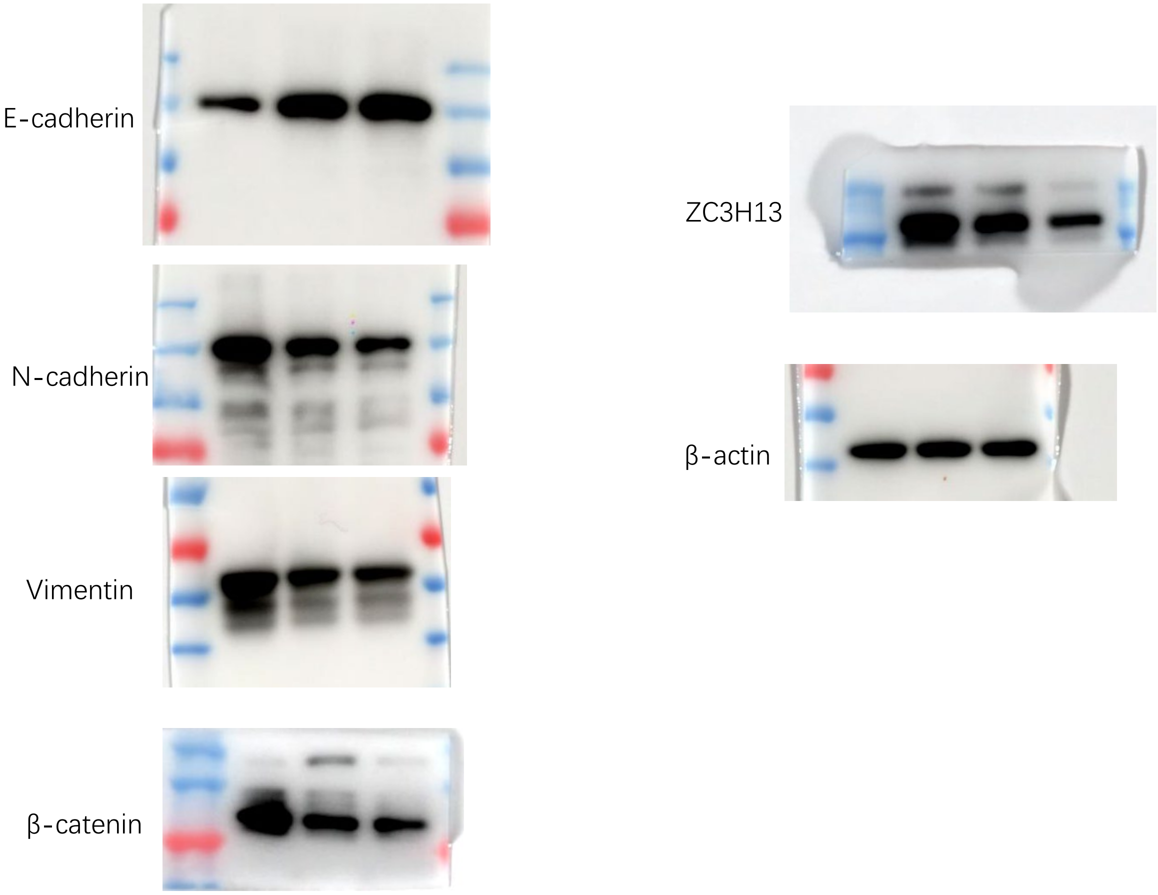

Figure 4H

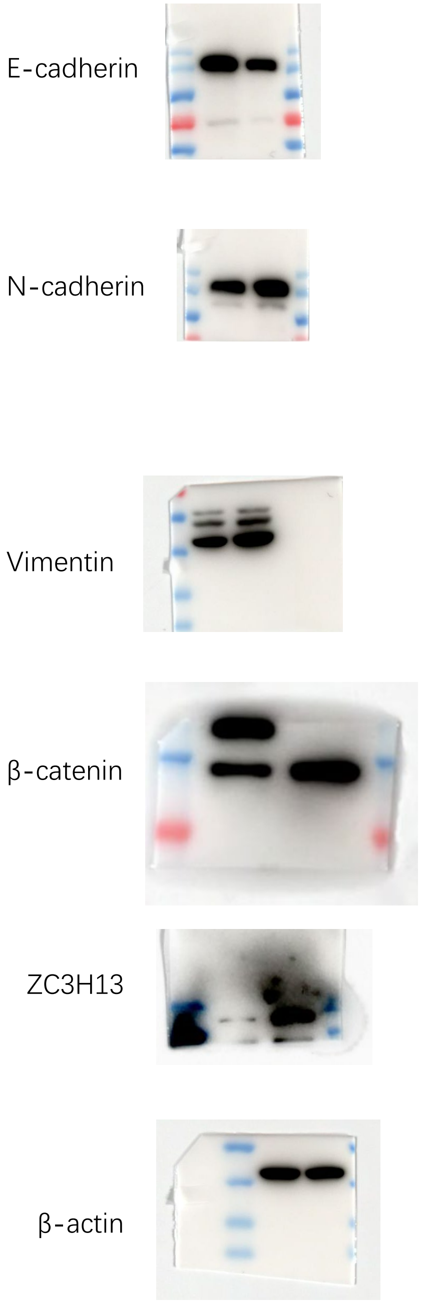

Figure 5F

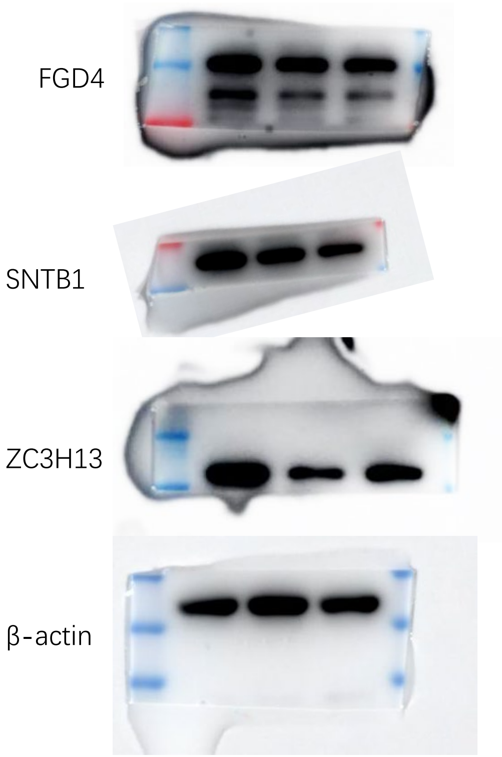

Figure 5H

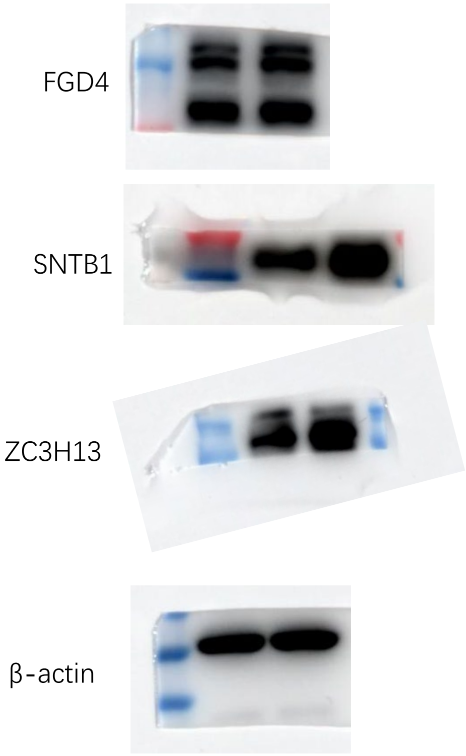

Figure 6A

YTHDF1

YTHDC2

IGF2BP1

IGF2BP2

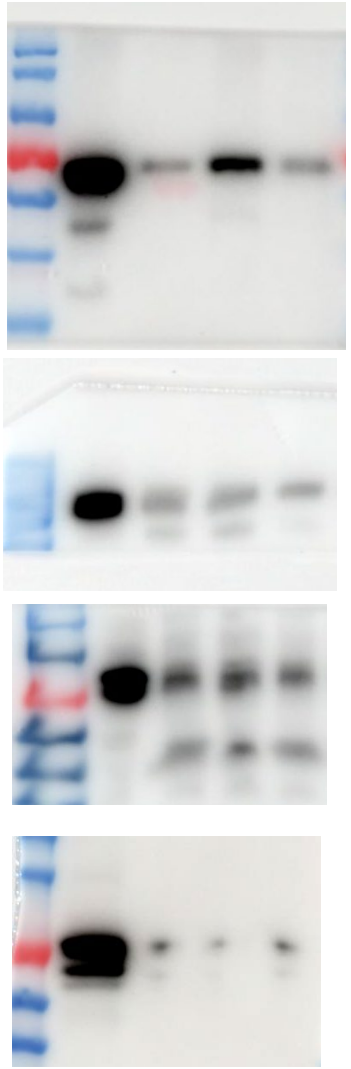

Figure 6J

E-cadherin

N-cadherin

Vimentin

β-catenin

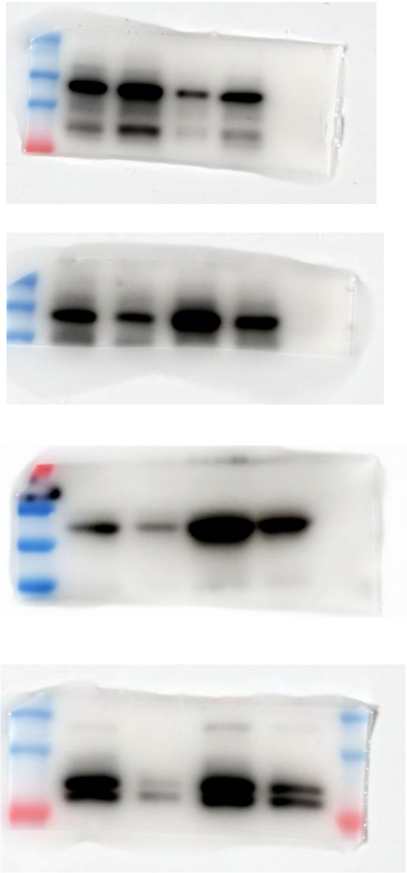

SNTB1

ZC3H13

β-actin

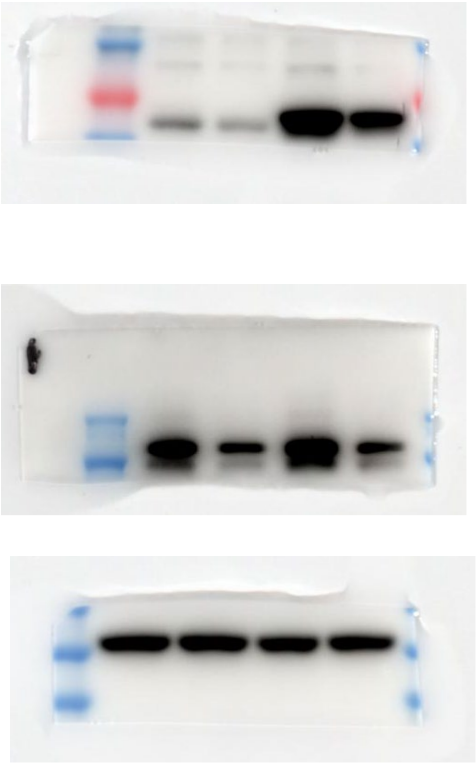

Figure 7F

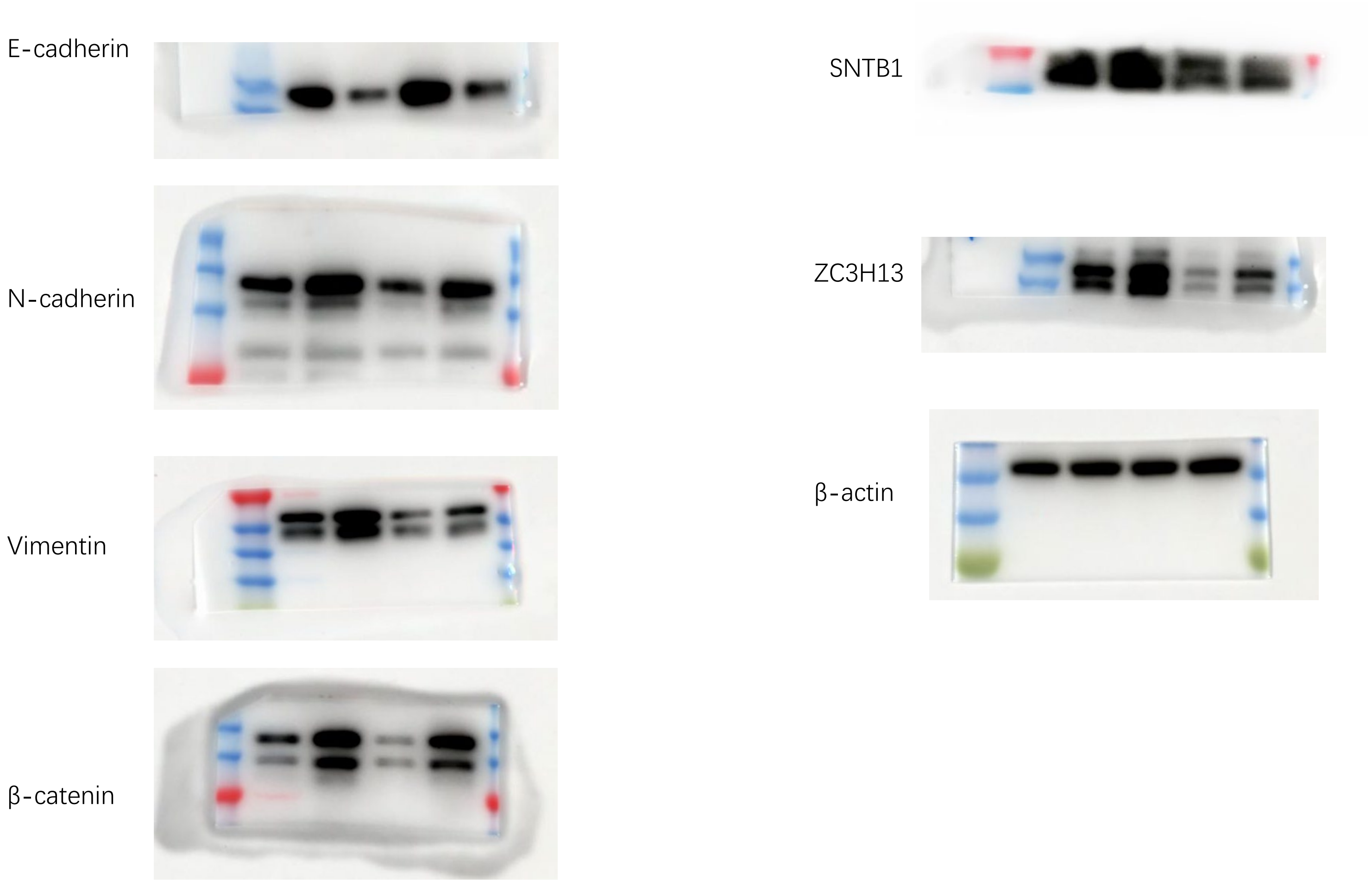
E-cadherin
N-cadherin
Vimentin
β-catenin
SNTB1
ZC3H13
β-actin

Figure S1B

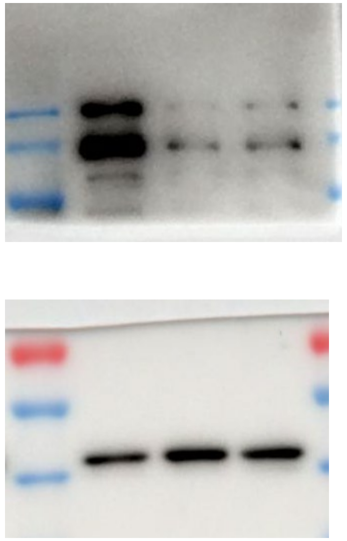

Figure S3

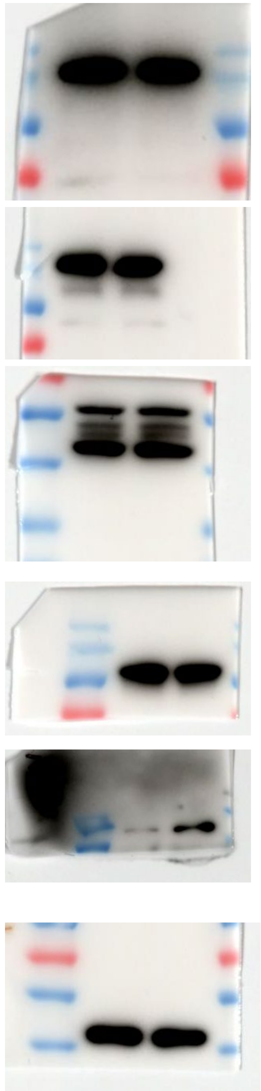

Figure S4B

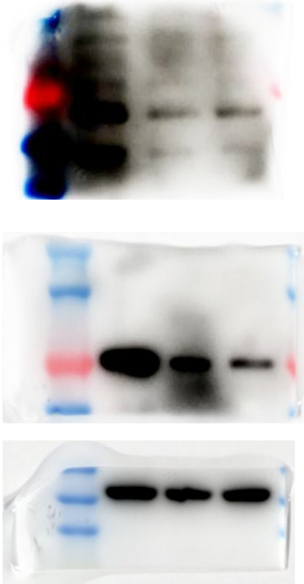

Figure S4C

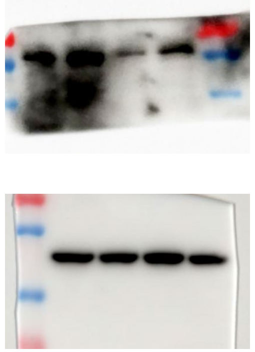

Supplement: Supplementary file 7 — Original western blot [file 41419_2025_7889_MOESM7_ESM.pdf]
